# Warburg’s Ghost—Cancer’s Self-Sustaining Phenotype: The Aberrant Carbon Flux in Cholesterol-Enriched Tumor Mitochondria via Deregulated Cholesterogenesis

**DOI:** 10.3389/fcell.2021.626316

**Published:** 2021-03-12

**Authors:** Peter S. Coleman, Risa A. Parlo

**Affiliations:** ^1^Independent (Retired) Academic, Beverly, MA, United States; ^2^Kingsborough Community College, Brooklyn, NY, United States

**Keywords:** Warburg effect, tumor cholesterogenesis, mitochondrial citrate export, truncated Krebs/TCA cycle, tumor membrane cholesterol

## Abstract

Interpreting connections between the multiple networks of cell metabolism is indispensable for understanding how cells maintain homeostasis or transform into the decontrolled proliferation phenotype of cancer. Situated at a critical metabolic intersection, citrate, derived via glycolysis, serves as either a combustible fuel for aerobic mitochondrial bioenergetics or as a continuously replenished cytosolic carbon source for lipid biosynthesis, an essentially anaerobic process. Therein lies the paradox: under what conditions do cells control the metabolic route by which they process citrate? The Warburg effect exposes essentially the same dilemma—why do cancer cells, despite an abundance of oxygen needed for energy-generating mitochondrial respiration with citrate as fuel, avoid catabolizing mitochondrial citrate and instead rely upon accelerated glycolysis to support their energy requirements? This review details the genesis and consequences of the metabolic paradigm of a “truncated” Krebs/TCA cycle. Abundant data are presented for substrate utilization and membrane cholesterol enrichment in tumors that are consistent with criteria of the Warburg effect. From healthy cellular homeostasis to the uncontrolled proliferation of tumors, metabolic alterations center upon the loss of regulation of the cholesterol biosynthetic pathway. Deregulated tumor cholesterogenesis at the HMGR locus, generating enhanced carbon flux through the cholesterol synthesis pathway, is an absolute prerequisite for DNA synthesis and cell division. Therefore, expedited citrate efflux from cholesterol-enriched tumor mitochondria via the CTP/SLC25A1 citrate transporter is fundamental for sustaining the constant demand for cytosolic citrate that fuels the elevated flow of carbons from acetyl-CoA through the deregulated pathway of cholesterol biosynthesis.

## Introduction

Since the 1970’s, sophisticated biochemical research tools and techniques have yielded a cornucopia of new detail on the molecular mechanisms of mitochondrial cellular bioenergetics, the individual pathway steps of intermediary metabolism, and the metabolic regulation of cell proliferation. The amassment of enzymological data on metabolic pathways, detailing complex enzymatic controls, spotlighted one of the fundamental and persistent metabolic controversies in the field of cell growth and proliferation—particularly the most clinically intractable obstacle: cancer.

Thus, over 50 years ago the debate re-emerged over which metabolic profile initiates or dictates the cancer cell phenotype, known for nearly 100 years worldwide as the Warburg vs. the Crabtree effect (Warburg, 1925; Crabtree, 1929).

Warburg’s original hypothesis, based on his careful tissue slice respiration measurements with the manometer apparatus he invented, posits that cancer cells, unlike normal tissue, derive the bulk of their ATP by means of glycolysis, a less efficient ATP-generating pathway, despite an abundance of systemic oxygen and the presumptive capacity for high yields of ATP via mitochondrial aerobic oxidative phosphorylation. Warburg’s aerobic glycolysis concept for tumors proposed that the aberrant metabolic profile in tumor cells was based on malfunctioning mitochondria. Crabtree, offered a different interpretation of Warburg’s theory of tumor metabolism. Based on similarly careful tissue slice respiration measurements in a variety of tumors, he proposed that in order to supply their ATP requirements, the high rate of metabolic flux through glycolysis actually depresses the tumor’s capacity for normal mitochondrial oxidative respiration via feed-back regulatory processes. Crabtree wrote, “The tentative conclusion is that glycolytic activity exerts a significant checking effect on the capacity for respiration of tumour tissue.”

It is remarkable that the Warburg-vs.*-*Crabtree-effect debate remains an open issue today. But, in Warburg’s era it wasn’t possible to detail any mitochondrial characteristics on a molecular level that might reveal their malfunction in cancer. And even with today’s more sophisticated techniques, if differences on the molecular structure-function level between normal and tumor mitochondria were indisputably documented, how could we determine whether such differences were a cause or consequence of the cancer phenotype? (Koppenol et al., 2011; Senyilmaz and Teleman, 2015; Potter et al., 2016).

The resurgent and ongoing interest in the Warburg effect makes clear that it is not an artifact of experimental conditions or selection of unique tissue subjects chosen for study (Cassim et al., 2020; Pascale et al., 2020). Originally, Warburg emphasized aerobic glycolysis and malfunctioning mitochondria as a causal or initiating factor of cancer (Warburg, 1956), and although this belief remains current (Seyfried, 2015), consensus of opinion today considers the Warburg effect to arise as a result of primary genetic mutations (Carter et al., 2009; Vogelstein et al., 2013; Lu et al., 2015).

Yet, in our view, despite the almost logarithmic increase in research publications on the Warburg effect and cancer over the last two decades (see Figure 1 in Otto, 2016), one fundamental characteristic of the tumor’s altered *overall* metabolic carbon flow pattern, involving cytosolic citrate, has not been addressed. While the cytoplasm’s access to and acquisition of citrate is clearly recognized as a central metabolite required by the tumor’s reorganized energy metabolomics and fatty acid synthesis, the mandatory role of citrate as precursor fuel for operation of the well-documented deregulated and enhanced carbon flux through the cholesterogenesis pathway in tumors has not been adequately recognized. This review hopes to emphasize a paramount link between mitochondrial bioenergetics in tumors and the select role(s) played by an increased membrane cholesterol content, which together help perpetuate the unrestrained cell proliferation phenotype of cancer.

## Purpose of This Review

Tumorigenesis is a relatively long-term and steady pathological process, phenotypically characterized by uncontrolled cell proliferation. Within recent decades, advanced understanding of the molecular details on the “unrestrained” growth of cancers has revealed a chameleon-like propensity for their metabolic malleability. Such profound metabolic complexity arises as a function of the timeline of differentiation from normal to neoplastic, the cell type, tissue, and even location within the particular tissue (Faubert et al., 2020). With regard to classic Warburg effect descriptors, as has been noted (Abdel-Haleem et al., 2017), transient rapid cell division, such as T-cell activation and angiogenesis, is still “regulated,” yet shares verifiable aerobic glycolysis features with “deregulated” tumor cell proliferation, and thus both display legitimate Warburg effect profiles. *No indisputably convincing argument has yet to identify, on a molecular level, first elements that become “deregulated” in the case of tumorigenesis, but remain “regulated” in transient cell proliferation.*

The purpose of this review is to reiterate our specifically focused perspective that:

(1) the Warburg effect’s proposal of an aberrant respiratory pattern in tumors can be tightly linked with the long-held, well-documented, deregulated, and enhanced cholesterol synthesis (Siperstein and Fagan, 1964; Chen et al., 1978; Heiniger, 1981; Coleman et al., 1997);
(2) the tumor cell’s membranes become enriched with cholesterol as a result of the well-evidenced enhanced rate of cholesterol biosynthesis in tumors;
(3) cholesterol enrichment of tumor mitochondrial membranes promotes and necessitates the continuous cytoplasmic supply of the precursor substrate (acetyl-CoA) for cholesterogenesis via the preferential export of citrate from mitochondria;
(4) implies, as others have, that communication or cross-talk between the plasma membrane Na^+^-dependent citrate transporter (PMCT, encoded by *SLC13A5*) and the mitochondrial inner membrane citrate transport protein (CTP, encoded by *SLC25A1*) might be critical to the proposed metabolic sequelae that largely define cell proliferation as the major phenotypic hallmark of cancer.

### Mitochondrial Metabolism Is Anomalous in Tumors

More than 30 years ago, inspired by an increasing abundance of exciting research implicating the pivotal role assumed by altered enzyme regulation of the cholesterol synthesis pathway in tumor cell proliferation, many laboratories (including ours) began to focus on the affect such a change in cholesterol biosynthesis would have on mitochondria as the cell’s main energy-generating machinery. As entry into the thrust of this review, the mitochondrial metabolomics involved in cholesterogenesis must be highlighted.

#### The Ins-and-Outs of Mitochondria: The Citrate Transporter (CTP/*SLC25A1*) Stands Out From the Crowd

The role of mitochondria is clearly of interest for two reasons. First, the stoichiometry of cholesterol biosynthesis requires 36 ATP per molecule, wherein mitochondrial oxidative phosphorylation would be the presumed major and most efficient ATP provider. Second, cytosolic acetyl-CoA is the initial precursor substrate of cholesterol (18 acetyl-CoA/cholesterol), whose multi-step synthesis is almost entirely localized on the endoplasmic reticulum (ER). In normal, oxygen-respiring cells amply supplied with glucose, the mitochondrion is a major cellular locus for glycolysis-derived acetyl-CoA, principally as a result of carbohydrate breakdown.

**FIGURE 1:**
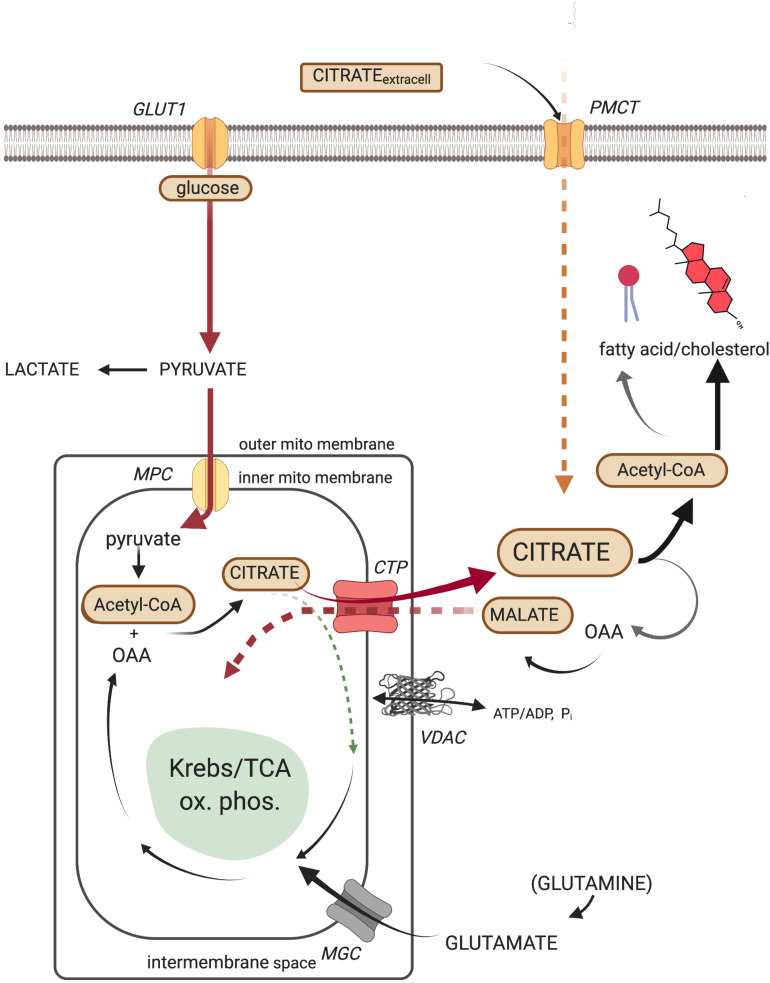
The cellular metabolic origin and fate of citrate. GLUT1, facilitated mammalian glucose transporter (SLC2A1); PMCT, plasma membrane citrate transporter (SLC13A5); MPC, mitochondrial pyruvate carrier (SLC54A2); CTP, citrate transport protein (SLC25A1); MGC, mitochondrial glutamate carrier (SLC25A22); VDAC, mitochondrial outer membrane voltage-dependent anion-selective channel.

Canonically, the source of the cytosolic acetyl-CoA required by all lipid synthesis is the key cellular metabolite, citrate. Recognition of the centrality of citrate as a cytoplasmic source of carbons in tumors and other proliferating cells cannot be overstated (Icard et al., 2012; Iacobazzi and Infantino, 2014). Citrate, ultimately formed from the catabolism of glucose to acetyl-CoA and its subsequent combination with oxaloacetate via the first step of the Krebs/TCA cycle, may be transported out to the cytosol via the well-studied mitochondrial citrate transport protein (CTP, the product of the *SLC25A1* gene) in a 1:1 exchange for the electroneutral import of another TCA intermediate, malate (Hanse et al., 2017). Malate, formed from the cytosolic cleavage of citrate, will shuttle back into the mitochondria via the CTP, in exchange for another exiting citrate, and once again become a TCA cycle participant. This metabolic routing of citrate, from mitochondria to cytosol, is the classic pathway utilized to generate, via ATP-citrate lyase enzymatic activity, the acetyl-CoA required for lipid anabolism. It must be noted, however, that environmentally stressful circumstances (e.g., hypoxia) provide a platform for the tumor to demonstrate its creative metabolic flexibility. Since, as mentioned, tumors develop in diverse and heterogeneous environments *in vivo*, the citrate that ultimately feeds lipid synthesis may arise from glutamine/glutamate as the non-glycolytic (non-pyruvate) carbon source (DeBerardinis et al., 2007; Wise et al., 2011; Mullen et al., 2014; Yang et al., 2014). Thus, the reductive carboxylation of α-ketoglutarate (a “reversal” in direction of the more traditional Krebs/TCA cycle carbon flux) by mitochondrial isoforms of NADP^+^/NADPH-requiring isocitrate dehydrogenase has also been shown to generate mitochondrial citrate, which then can become available to the cytosol. Human hepatomas, whose mitochondria are cholesterol enriched, have been shown to exhibit such an altered metabolite flow, through participation of the selective over-expression of the α-ketoglutarate transport protein (*SLC25A11*) (Baulies et al., 2018). Ultimately, it is recognized that by whatever metabolic manipulations the tumor’s creativity elicits, supplying the cytosolic pool with citrate occupies the metabolic center of gravity for lipid biosynthesis (Figure 1).

But, wait! The operation of the conventional Krebs/TCA cycle (i.e., in normal, non-proliferating-cell mitochondria) is usually appreciated first, without considering the interplay of the numerous metabolite and ion transport proteins embedded in the mitochondrial inner membrane that allow communication with the cytosol (LaNoue and Schoolwerth, 1979; Palmieri, 2013). Participation of these membrane metabolite transport proteins simultaneous with the operation of the Krebs/TCA cycle, when comparing normal vs. pathological metabolism, complicates the metabolic reprogramming considerably!

For example, are we to believe that all (more than 50 in humans; Palmieri and Monné, 2016) inner mitochondrial membrane metabolite transporters operate independent of any sort of regulatory influence, oblivious to moment-to-moment cellular demands, such as the dividing cell’s temporal position within the cell cycle? What metabolic environmental condition(s) in the cell could serve as signals that would control regulation of metabolite flux between mitochondrial matrix and cytosol?

#### Loss of Feed-Back Control of Cholesterogenesis in Tumors: Evidence and Some Consequences

By 1964 (Bloch, 1965) there was already reasonable suspicion that regulation of cholesterogenesis in animals centered on modulating the activity of the ER-bound enzyme 3-OH-3-CH_3_-glutaryl-CoA-reductase (HMGR). Subsequent research leaves little doubt that inhibition of HMGR activity, and the resulting lack of cholesterol synthesis, suppresses cell division. Especially provocative are two findings (Chen et al., 1975; Brown and Goldstein, 1980; Heiniger, 1981; Doyle and Kandutsch, 1988).

First, the flow of anabolic carbons in the cholesterogenesis pathway, specifically the genesis of mevalonate, the product of the HMGR reaction, serves an indispensable role in initiating DNA synthesis and cell proliferation. In fact, the addition of mevalonate to circumvent a blocked HMGR activity re-establishes cell growth (Sinensky and Logel, 1985). Second, and even more relevant: the loss of feedback inhibition of HMGR, and a resulting increase in HMGR activity, is a fundamental metabolic defect of virtually all cancers (Siperstein and Fagan, 1964; Goldstein and Brown, 1990). The overwhelming conclusion of the collective data from diverse laboratories, beginning in the 1980’s, reveals that cholesterogenesis in tumor cells not only lacks feedback regulation, but, depending on the rate of cell proliferation, can occur at very high, continuous rates. At first glance these findings would evince little surprise. After all, proliferating cells require newly replicated “everything”—the whole panoply of membrane lipids, including the membrane insertion of lipid bilayer fluidity-reducing cholesterol, the unique membrane-stabilizing sterol component in mammals (Demel and De Kruyff, 1976; Marquardt et al., 2016).

A penetrating question emerges. What global, as well as intracellularly specific, consequences can such abnormally high rates of cholesterogenesis reveal in proliferating tumors? Tumor cholesterol overproduction implies that the various cellular membrane categories (plasma, ER, etc.) might become differentially cholesterol-enriched, in some cases incorporating enlarged lipid-raft domains. Such enrichment might alter the phase-transition properties of membrane lipids, thereby modifying the functional characteristics of integrated membrane-associated proteins (Siperstein, 1984; Liu et al., 2017). Would differential cholesterol enrichment of tumor membranes have meaningful consequences for the tumor’s metabolomics and growth?

Siperstein’s (1984) comprehensive review coincided with our own laboratory’s ongoing investigations that sought correlations between the standard, respiration-linked oxidative phosphorylation patterns of normal liver, and those exhibited by the various cholesterol-enriched Morris hepatoma model systems. Specifically, we confirmed that the cholesterol content of isolated mitochondria generally parallels the growth rate of these hepatomas; faster growing tumors (e.g., Morris hepatoma 3924A) display substantially higher mitochondrial cholesterol than the mitochondria of slower growing tumors (e.g., Morris hepatoma 16), and both hepatomas possess higher membrane cholesterol than normal (or host rat) liver mitochondria. Indeed, the cholesterol enrichment of tumor mitochondrial membranes was then, and continues to remain, familiar to investigators (van Hoeven and Emmelot, 1972; Feo et al., 1975; Chan and Barbour, 1983; Epand, 2006).

#### The Road to Mitochondrial Membrane Cholesterol Enrichment

Metabolically speaking, the mitochondrial double membrane architecture gives rise to a division of labor. The matrix-facing inner membrane is responsible for a variety of mitochondrial functions including the entire O_2_-requiring, Krebs/TCA cycle-linked bioenergetics enterprise, pregnenolone synthesis, and the operation of the more than 50 substrate transport protein complexes in at least 25 subfamilies embedded in the lipid bilayer, such as the CTP/*SLC25A1* transport protein (Palmieri, 2013; Palmieri and Monné, 2016). The outer mitochondrial membrane, the lipid bilayer barrier that separates the cytosol from the space between both mitochondrial membranes, contains the voltage-dependent anion channel (VDAC) as its most abundant protein (Colombini, 2004). Along with VDAC, other associated outer membrane protein moieties participate in regulating molecular traffic between cytosol and matrix (Campbell and Chan, 2008). VDAC is considered the fundamental control channel that regulates transport of ADP/ATP and other ions and metabolites through the outer membrane barrier into the inter-membrane space (Hiller et al., 2010; Shoshan-Barmatz et al., 2015). Within this inter-membrane space substrates are positioned proximal to the multiple transporters of the inner membrane which then transmit them to the enzymatic machinery of the matrix. VDAC’s high-resolution structure has been established (Camara et al., 2017), and most compellingly, has been shown to bind cholesterol (Hulce et al., 2013).

Depending on the tissue in normal, non-proliferating cells, the cholesterol content of the inner mitochondrial membrane is poor compared with its outer, cytosol-facing membrane. Indeed, relative to other cell membranes, cholesterol is a minor lipid component of both mitochondrial membranes (Horvath and Daum, 2013). Nevertheless, there is general accord that cholesterol’s presence in mitochondrial membranes influences mitochondrial metabolic function (Rostovtseva and Bezrukov, 2008; Martin et al., 2016). Despite the relatively modest cholesterol content of mitochondria compared with the plasma membrane, HeLa cell mitochondria have been shown to possess cholesterol/sphingolipid-rich (lipid raft) microdomains (Mollinedo et al., 2011). The well-established cholesterol enrichment of tumor mitochondria (Kaplan et al., 1982; Crain et al., 1983; Parlo and Coleman, 1984, 1986; Coleman et al., 1997) and its potential effect on the topology of lipid rafts and the fluidity of laterally surrounding phospholipids, might logically alter interactions between membrane-integral proteins, thereby yielding tumor-specific carbon flux patterns (Marquardt et al., 2016; Liu et al., 2017).

However, the intracellular route by which cholesterol reaches the CTP membrane environment may impact cholesterol’s influence on the operation of the CTP. Cholesterol must be conveyed from its source of origin, then delivered from those intracellular membrane loci external to mitochondria, across the mitochondrial inter-membrane space, to the inner membrane (Liu et al., 2006; Flis and Daum, 2013). The vehicle primarily responsible for delivering cholesterol to the inner mitochondrial membrane is the steroidogenic acute regulatory protein (StAR/STARTD1), although its precise mechanism of cholesterol delivery to the inner membrane is not fully established (Martin et al., 2016; Elustondo et al., 2017). StAR/STARTD1-mediated cholesterol transport to the inner membrane is the rate-controlling step for all steroid hormone biosynthesis, and its over-expression has been proposed to correlate with enhanced delivery of cholesterol in breast cancer (Manna et al., 2019). Nevertheless, any potential effect on the activity of the CTP due to enhanced cholesterol delivery to the inner mitochondrial membrane by StAR/STARTD1 remains conjectural. Clinical evidence, however, indicates that mutations in the CTP gene can promote serious neuronal disfunction (Chaouch et al., 2014), so it is plausible that transmittal of excess cholesterol to the inner membrane by over-expressed StAR/STARTD1, and a resulting cholesterol-rich, laterally distorted lipid bilayer, could effect a significantly altered CTP activity (Demel and De Kruyff, 1976; Mollinedo et al., 2011; Marquardt et al., 2016).

In order to confront this possibility we reasoned that one of the most appropriate measurable functions to correlate with the extent of such mitochondrial cholesterol enrichment would concern the penultimate source of substrate for the tumor’s deregulated cholesterogenesis: the citrate that shuttles between the mitochondrial matrix and the cytosol on the CTP (Gnoni et al., 2009). Indeed, contemporary data (Catalina-Rodriguez et al., 2012) indicate mitochondrial CTP levels are increased in several cancers, while oncogenic p53 mutants stimulate CTP expression and promote tumor cell proliferation (Kolukula et al., 2014).

#### Cholesterol-Enriched, Isolated Hepatoma Mitochondria Preferentially Export Citrate

As a reminder, the CTP in normal liver mitochondria catalyzes the electroneutral exchange across the mitochondrial inner membrane of citrate for either another tricarboxylate, a dicarboxylate (e.g., malate or succinate), or phosphoenolpyruvate (Palmieiri et al., 1972; LaNoue and Schoolwerth, 1979). Using the specific CTP inhibitor, 1,2,3-benzenetricarboxylate (BTC), Kaplan et al. (1982) completed the first comprehensive study on the kinetic characteristics of CTP exchange transport in tumor vs. normal mitochondria, demonstrating that a highly probable positive correlation exists between cholesterol enrichment of tumor mitochondria and an increased V_*max*_ for citrate transport displayed by the CTP.

**FIGURE 2:**
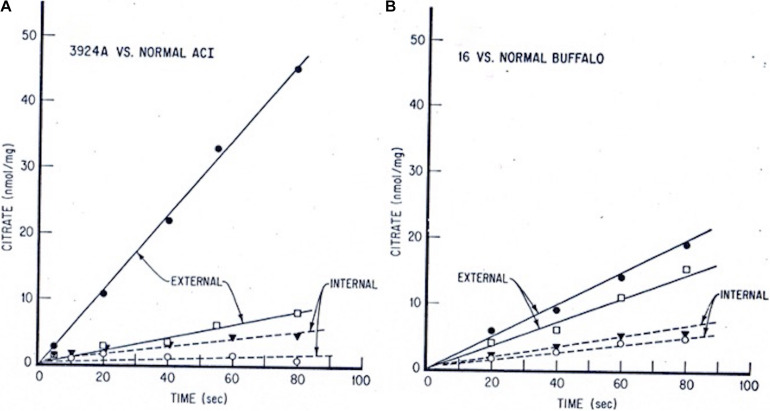
Tumor vs. normal liver extra- and intra-mitochondrial citrate levels: time-course incubations fed pyruvate + malate. Mitochondria from each tissue source were incubated with 0.5 mM pyruvate/0.1 mM malate/15 mM ADP. At indicated time intervals, incubation aliquots were rapidly centrifuged through a silicone oil layer into perchloric acid. Extramitochondrial citrate was determined on the samples above, and intramitochondrial citrate was determined on samples below the silicone oil barrier. **(A)** Hepatoma 3924A, • extramito; ○ intramito. Normal ACI rat liver, □ extramito; ▼ intramito. **(B)** Hepatoma 16, • extramito; ○ intramito. Normal Buffalo rat liver, □ extramito, ▼ intramito (see: Parlo and Coleman, 1984, for details on methods).

**FIGURE 3:**
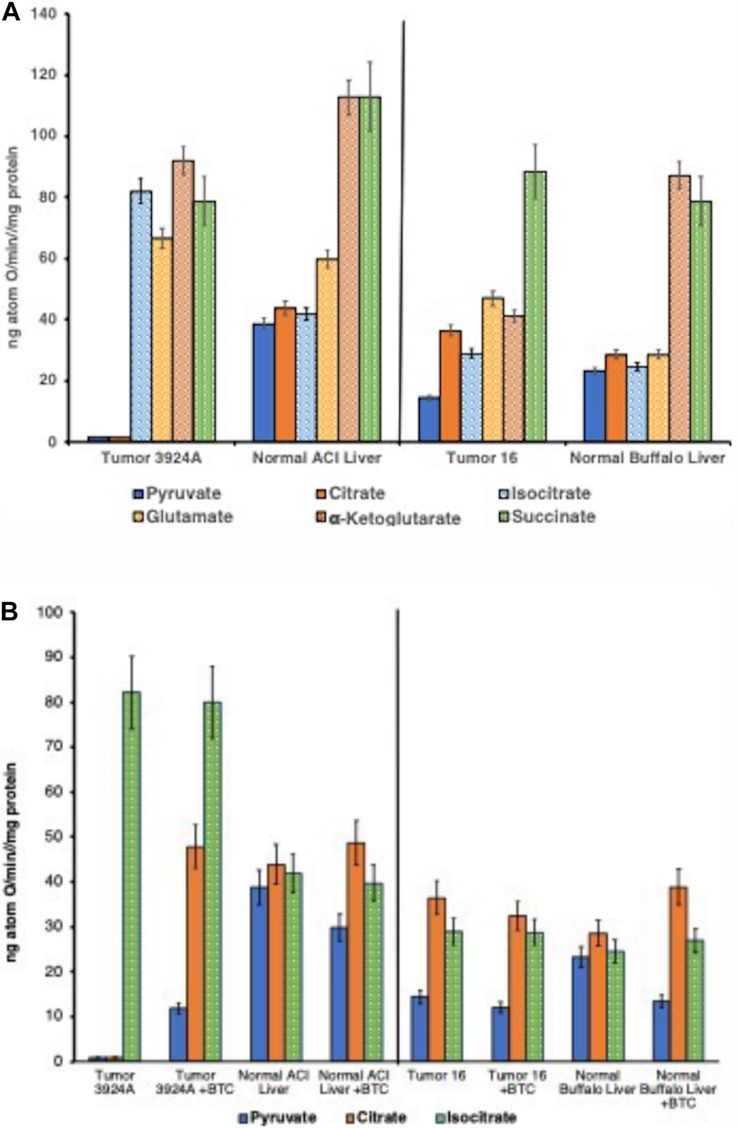
**(A)** State 3 rates: O_2_ consumption tumor vs. normal mitochondria. Duplicate incubations (30°C) contained 5 mM respiratory substrates *plus* 250 μM ADP addition aliquots. ADP/O ratios for all NAD^+^-linked substrates averaged 2.63; for succinate, 1.77. ADP/O ratios were unmeasurable with mitochondria from Morris hepatoma 3924A when fueled by pyruvate or citrate since ADP-initiated O_2_ uptake was negligible. Data error bars are means ± SD. **(B)** State 3 rates: O_2_ consumption pre-/post-aconitate hydratase, tumor vs. normal mitochondria, ± BTC. Incubations were performed as described for **(A)**. 150 μM BTC additions to each incubation were made 30 s after substrate and 30 s prior to additions of 250 μM ADP. Data error bars are means ± SD.

Corroborating evidence came from our laboratory’s continuing experiments with the wider, more globally accessible metabolic respiratory profiles of normal liver, slow- and rapidly growing Morris hepatoma mitochondria. We documented the more than 4-fold faster pyruvate-supplied citrate efflux from the fast-growing, highly (>5-fold) cholesterol-enriched hepatoma 3924A mitochondria relative to their normal liver counterparts. Both intra-mitochondrial and external-milieu citrate levels were assayed periodically on aliquots of actively respiring mitochondria from the rapid (hepatoma 3924A) and slow (hepatoma 16) growing tumors vs. their control livers over an extended incubation time-course (Figures 2A,B). Strikingly, the accelerated, abbreviated carbon flux into and out of the tumor mitochondria (pyruvate_*cyto*_— > pyruvate_*mito*_— > acetyl-CoA_*mito*_— > citrate_*mito*_—> [CTP]— > citrate_*cyto*_) was accompanied by a negligibly small ADP-initiated O_2_ uptake (Figure 3A). These findings implied that the cholesterol-rich tumor mitochondria selectively ejected the Krebs/TCA cycle-generated citrate to the external milieu, rather than employing it to fuel respiration-linked oxidative phosphorylation. Slow-growing (“minimally deviated”) Morris hepatoma 16 mitochondria elicited a similar, abbreviated citrate carbon efflux, albeit much reduced in proportion to their lower-level cholesterol enrichment (∼2-fold) compared with normal liver mitochondria (Parlo and Coleman, 1984).

Yet, this conspicuously aberrant tumor mitochondrial respiratory pattern that centered on the re-routing of citrate from the TCA cycle to the cytoplasm, was not detected when these cholesterol-rich mitochondria were fueled with substrates joining the TCA cycle beyond citrate (i.e., post the aconitate hydratase step). That is, although virtually no ADP-stimulated O_2_ uptake occurred when supplied with either pyruvate or citrate, the cholesterol-rich tumor mitochondria respired almost indistinguishably as well as normal when fueled with substrates beyond citrate in the TCA cycle sequence: viz., isocitrate, α-ketoglutarate, succinate, and even with glutamate (Figure 3A; Parlo and Coleman, 1984; Kaplan et al., 1986).

Validation of this deviant mitochondrial respiratory carbon flux was further confirmed by blocking the hepatoma CTP with the selective inhibitor BTC, which remarkably (but expectedly) reversed the nearly absent respiration of pyruvate-fed O_2_ uptake (Figure 3B). These results appeared to us as striking and far-reaching. Forcing citrate to remain in the TCA cycle by blocking its export to the cytosol allowed for the re-establishment of near-normal oxidative phosphorylation with cholesterol-enriched organelles from both fast and slow-growing tumors. The significance, here, establishes among other things, that the hepatoma mitochondria possess perfectly functional aconitate hydratase activity, and apart from their membranes being abnormally cholesterol-enriched, these tumor mitochondria proved eminently capable of performing the oxidative phosphorylation acrobatics of their normal counterparts, if matrix-generated citrate were prevented from exiting.

In this regard it is worth considering that early studies with isolated mitochondria from a variety of tumors (Aisenberg, 1961) displayed lower respiratory rates specifically fueled with pyruvate or citrate compared with corresponding normal mitochondria. Yet, because all tumor mitochondria tested since then have been shown to possess at least some capacity to respire with a number of alternative substrates (succinate, glutamate, even fatty acid derivatives like β-hydroxybutyrate—see for ex., Table 5, Aisenberg, 1961), the respiratory ability of tumor mitochondria in general was taken to be fundamentally normal, or at least functionally unexceptional, and allusion to Warburg’s hypothesis unnecessary of discussion in this context.

#### Technical Controversies Regarding the Exogenous Enrichment of Normal Mitochondria With Cholesterol

Experimental approaches to the same or similar questions can often vary from one laboratory to another, especially as new technologies are applied. Explicitly presented methods, carefully followed and reproduced by different laboratories, become the “gold standard” by which the global veracity of experimental results are confirmed and established. However, deviation from one laboratory’s carefully specified methods, regardless of how seemingly inconsequential, will often yield results by the second that differ from the first. Thus, contradictory conclusions about fundamental mechanisms based upon data derived by different methods, and/or relying on a single tumor system, can be misleading, at best!

Mindful of these considerations, the striking results seen on accelerated mitochondrial CTP-facilitated citrate transport in both slow- and rapidly growing rat hepatomas relative to normal liver organelles, encouraged consideration that there was a direct, positive correlation between the extent of mitochondrial cholesterol enrichment and citrate export, as described above. Would normal liver mitochondria, if purposefully enriched with cholesterol, mimic the behavior of tumor mitochondria with respect to their handling of citrate?

A variety of diverse methods have been employed over many years to alter the membrane cholesterol content of viable cells in experimental animals (Colell et al., 2003; Solsona-Vilarrasa et al., 2019) including relatively long-term dietary modification (Feo et al., 1975). Our laboratory had experimented with an early version of what we termed a solid-phase transfer method, described in detail, to covalently label the plasma membrane of viable human lymphocytes in suspension (Coleman et al., 1978a), employing Sephadex G-10 beads. After extensive further tests, we applied modifications of this solid-phase method that were able to successfully increase cholesterol levels in isolated normal mitochondria (see: Parlo and Coleman, 1984; **Supplementary Material**, for detailed methods; Coleman et al., 1978b; Coleman and Lavietes, 1981). This method reproducibly permitted the incremental titration of different amounts of cholesterol into the organelles. Yet, critically, such modified mitochondria were shown to retain full respiratory functional integrity.

**FIGURE 4:**
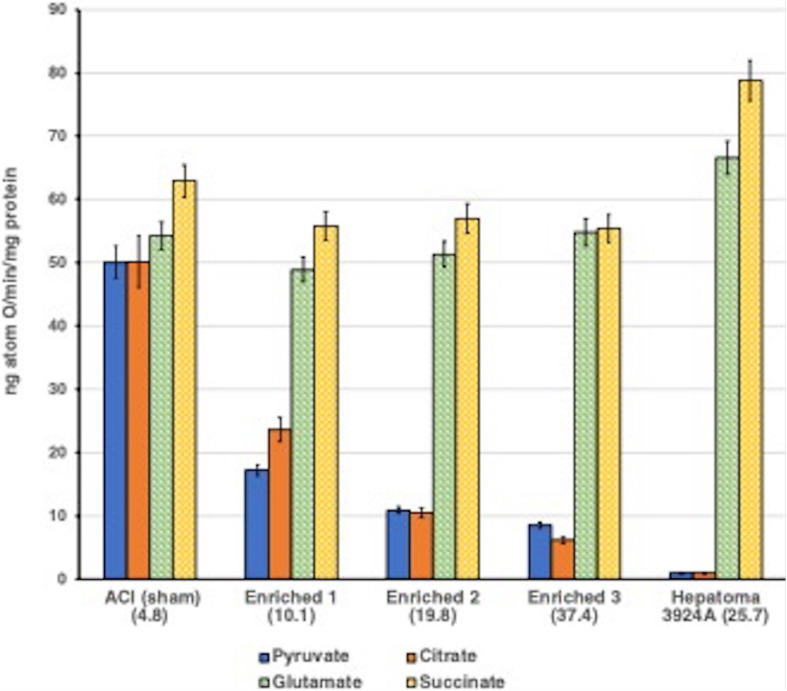
Aberrant pyruvate- and citrate-fueled O_2_ consumption by normal mitochondria exogenously enriched with cholesterol. Incubations were performed as described for Figures 3A,B. See text. For cholesterol-loading methodology with normal ACI liver mitochondria (see Parlo and Coleman, 1984). Data error bars are means ± SD. ACI (sham) indicates normal mitochondria treated by the cholesterol loading procedure, but without cholesterol. Normal mitochondria were enriched with three incremental cholesterol levels. The cholesterol content of each enrichment is shown as (μg total cholesterol/mg protein) in parentheses. Inverse correlation between the decrease in pyruvate- and citrate-fueled respiration as the cholesterol enrichment of normal mitochondria is exogenously increased is clear, dramatic and mirrors the respiratory pattern of tumor 3924A mitochondria. Note that respiration fueled by post-aconitate hydratase Krebs/TCA cycle substrates appears to be unaffected by the mitochondrial cholesterol content.

The results obtained after exogenous cholesterol enrichment of normal mitochondria, titrated to three increasing levels of cholesterol relative to control, mimicked remarkably both the re-routing pattern of CTP-promoted citrate export, and, in like manner, altered respiratory-linked oxidative phosphorylation observed with naturally occurring cholesterol-rich hepatoma mitochondria (for details see: Parlo and Coleman, 1984). Correspondingly, the cholesterol-loaded normal liver mitochondria revealed a 2-fold increase in the rate of pyruvate-fed citrate efflux, and a lower intramitochondrial steady-state citrate level compared with control mitochondria, again corroborating the preferential export of citrate observed with the tumor mitochondria (Figure 4).

Studies from one other laboratory, limited to isolated AS-30D hepatoma mitochondria, yielded contrary results based either on flawed application of, or incompletely defined, experimental methodology and inadequate data interpretation (Dietzen and Davis, 1993, 1994). To date, these unique and contradictory reports have never been validated by other laboratories. There are overwhelming data from numerous laboratories acquired over many years that speak specifically to the functional effects of altered mitochondrial membrane cholesterol (Schneider et al., 1982; Weiser et al., 2014). A wealth of research clearly and repeatedly demonstrates that cholesterol levels in mitochondrial membranes contribute to that organelle’s increasingly divergent metabolic function, by not only distorting cell maintenance mechanics, but also by affecting the process of cellular apoptosis (Colell et al., 2003; Tait and Green, 2012; Kennedy et al., 2014; Ribas et al., 2016).

#### Tracking Carbon Flux With Liver vs. Hepatoma Tissue *ex vivo*: What Happens to Pyruvate-Derived Citrate?

The well-established inability of tumors to demonstrate tight feedback control over cholesterogenesis suggests that the carbon flux through the multistep cholesterol biosynthesis pathway might operate continuously in tumors if amply supplied with acetyl CoA (Chen et al., 1978; Heiniger, 1981; Fairbanks et al., 1984; Mountford et al., 1984; Maltese and Sheridan, 1985; Erickson et al., 1988). Over a prolonged time-course therefore, buildup of cholesterol would be expected to be greater in tumors than in normal tissue, depending on the extent of the synthetic pathway’s impairment of the HMGR rate-limiting step (Rostovtseva and Bezrukov, 2008; Marquardt et al., 2016).

Results outlined above with isolated tumor and cholesterol-loaded normal mitochondria (Parlo and Coleman, 1986) motivated further investigation of the preferential export of mitochondrially generated citrate, but under more biologically realistic, whole cell conditions, comparable to those employed by Warburg, viz., viable tissue slices from normal liver and Morris hepatoma 3924A.

The primary objective of these comparative *ex vivo* incubation studies was to track the fate of [U-^14^C]-pyruvate carbons by following its metabolic conversion to both ^14^CO_2_ and [^14^C]-cholesterol. These data emerged, significantly, as two recognizably distinct carbon flux patterns. Normal and tumor systems were distinguishable by the preferential routes each tissue source used to metabolize the exogenous pyruvate; either *via* TCA cycle-linked oxidative decarboxylation or *via* anabolic cholesterogenesis (Parlo and Coleman, 1984).

**FIGURE 5:**
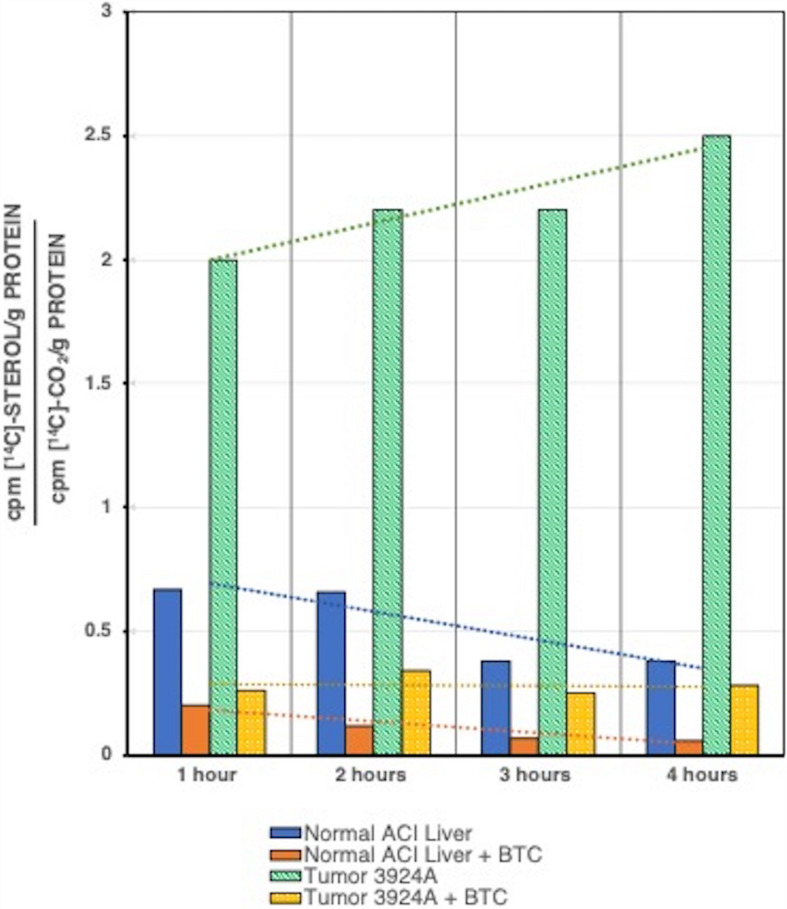
[U-^14^C]Pyruvate incorporation into [^14^C]cholesterol or ^14^CO_2_ for tumor 3924A vs. normal liver tissue slice incubations, ± BTC. Normal liver and tumor tissue slices were incubated with [U-^14^C]pyruvate *plus* or *minus* 10 mM BTC, and assayed at each time interval shown. Data are given as ratios over time for ^14^C incorporated into sterol vs. CO_2_. In addition to the clearly enhanced rate of sterol synthesis in tumor 3924A, the trend lines over time indicate that in this rapidly growing hepatoma, whose mitochondria are highly enriched in membrane cholesterol, BTC dramatically reverses the pyruvate carbon flux from sterol incorporation to CO_2_ formation (see: Parlo and Coleman, 1986 for details on methods).

Relative carbon flux ratios demonstrated clearly that after a 2-h incubation the hepatoma 3924A tissue slice system incorporated greater than 3-fold more [^14^C] into cholesterol than into ^14^CO_2_ compared with normal liver, and after a 4-hr incubation greater than 6-fold more [^14^C] incorporation appeared in cholesterol than in ^14^CO_2_, relative to normal liver. Most significantly, the specific mitochondrial CTP inhibitor, BTC, was capable of dramatically blocking [U-^14^C]-pyruvate-to-[^14^C]-cholesterol incorporation in the tumor tissue, effectively restoring a carbon flux pattern to one closely resembling the respiratory oxidative decarboxylation exhibited by normal liver tissue (Figure 5).

In later studies, described in section “Resurrecting the Truncated Krebs/TCA Cycle and the Warburg Effect.” subsection “BTC Inhibition of CTP Arrests (Reversibly) Cholesterol Synthesis and DNA Replication in Synchronized Tumor Cells,” below, the BTC uptake rate was directly measured and was confirmed to rapidly enter proliferating, cultured murine lymphoma cells during *in vitro* incubation.

Comprehensive evaluation of these metabolic patterns from normal and tumor sources, employing both isolated mitochondria and viable *ex vivo* tissue slices, supports the following general conclusions:

(1) When mitochondria of hepatomas become enriched with cholesterol by means of accelerated cholesterogenesis due to loss of feedback regulation of HMGR, they manifest an altered metabolic profile that exhibits a preferential export of citrate.
(2) Tumor mitochondria that are cholesterol enriched evince a significantly increased V_*max*_ for the mitochondrial membrane CTP.
(3) Preferential citrate efflux from the experimental tumor mitochondria deprives the conventional Krebs/TCA cycle of this critical respiratory substrate for oxidative phosphorylation-linked ATP generation regardless of ambient oxygen levels.
(4) The specific CTP inhibitor, BTC, blocks the preferential efflux of citrate from cholesterol-rich tumor mitochondria, thereby restoring citrate-fueled respiration and re-establishing the Krebs/TCA cycle respiration-linked oxidative phosphorylation process of normal mitochondria.
(5) The ability to support respiration-linked oxidative phosphorylation fueled by alternative substrates joining the Krebs/TCA cycle beyond the formation of citrate appears unaffected by the enhanced rate of citrate export from cholesterol-rich tumor mitochondria.
(6) The ratio of mitochondrial citrate to cytosolic citrate in the cholesterol-rich tumor mitochondria may be a critical expression of cell proliferation in cancer.

#### Accelerated, De-Regulated Cholesterogenesis Is Reinforced by the Multifold Expression of HMGR in Tumors

Although the regulation of cholesterol biosynthesis in normal cells is effected by means of several highly complex mechanisms, including feedback inhibition of HMGR by mevalonate-fueled pathway intermediates, hydroxysterols and a diverse host of other cellular metabolites (Goldstein and Brown, 1990), one could question whether the same set of controls obtains in tumors (Erickson et al., 1988). On the basis of the cumulative results described above with normal liver vs. rapidly growing Morris hepatoma 3924A, and since HMGR is a membrane-integral protein of the ER, we questioned whether the significantly increased carbon flux from pyruvate to cholesterol in the tumor could be detected in a cell-free, post-mitochondrial supernatant system (PMS) incubation. Moreover, if detectable, would such cell-free, post-mitochondrial HMGR activity retain any capacity for regulation? Finally, we asked whether an enhanced carbon flux in the tumor PMS compared with that of normal liver depended on the expressed cellular amount of HMGR.

**FIGURE 6:**
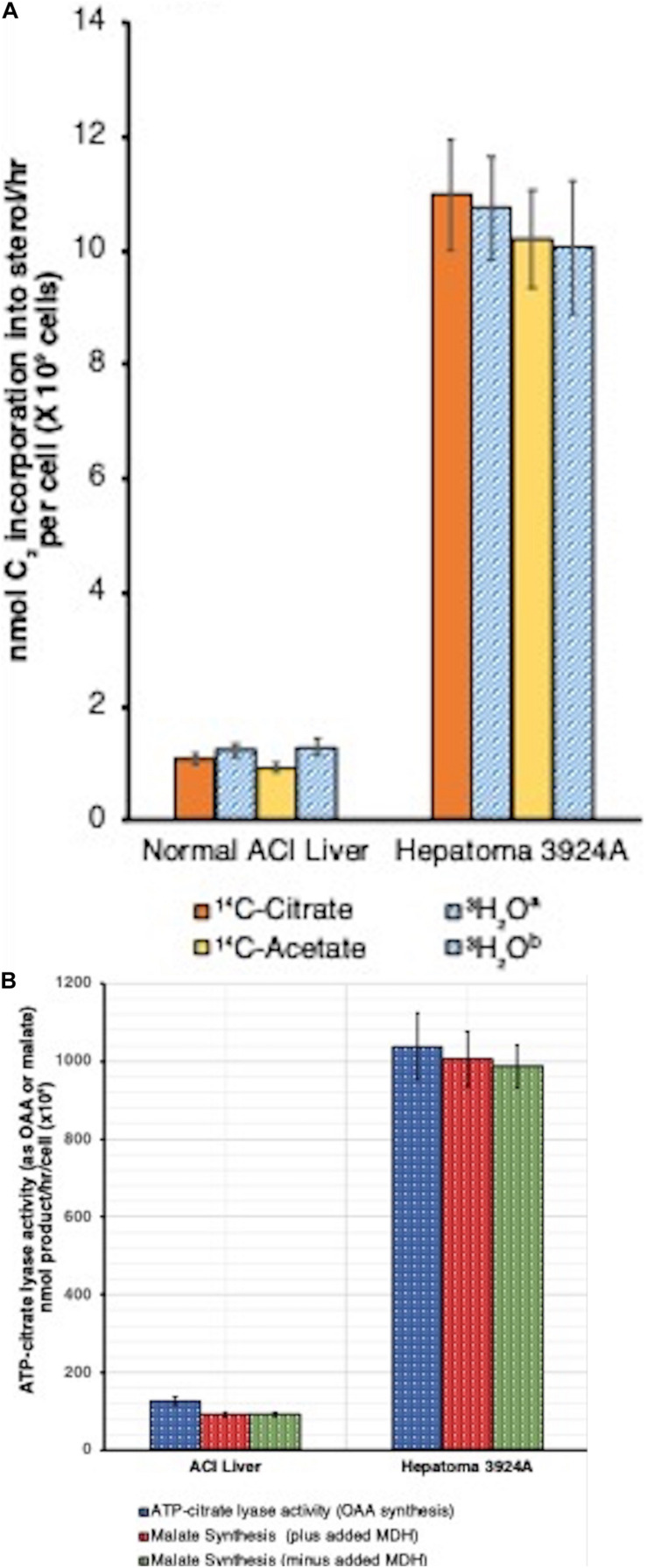
**(A)** Cell-free (post-mitochondrial) sterol synthesis rates, tumor 3924A vs. normal liver. Post-mitochondrial system (PMS) incubations (4 h, 37°C) contained either [1,5-^14^C]-citrate or [1-^14^C]-acetate (1.5 μCi/ml, 10 mM), or 10 mM citrate**^*a*^** or acetate**^*b*^** in the presence of [^3^H]-H_2_O (5 mCi/ml). Data error bars are means ± SEM. A correction factor of 1.5 was required for calculation of C_2_ incorporation into sterol from [1,5-^14^C]-citrate or [1-^14^C]-acetate because only 12 of the 18 C_2_ units supplied are incorporated/mol cholesterol. With the [^3^H]-H_2_O incubations, a correction factor of 0.536 was used, equivalent to the (^3^H/C) incorporation ratio into cholesterol (14-to-15 [^3^H]/27 cholesterol carbons) (see: Azrolan and Coleman, 1989, for details on methods). **(B)** Cell-free (post-mitochondrial) ATP-citrate lyase activity, tumor 3924A vs. normal liver. Data from two complementary procedures on PMS incubation aliquots (4 h, 37°C) are shown **(1)**. Direct ATP-citrate lyase activity (measured as oxaloacetate, OAA formed), and **(2)** anion exchange-resolved malate generated (*plus* and *minus* added malate dehydrogenase, MDH, 2 units/ml, to assure reaction completion). Data error bars are means ± SEM (see: Azrolan and Coleman, 1989, for details on methods).

Our study (Azrolan and Coleman, 1989; Coleman and Sepp-Lorenzino, 1990) demonstrated the first, to our knowledge, mitochondria-free cell lysate (PMS) capable of lipid synthesis. In fact, the capacity of the PMS for cholesterol synthesis fueled by [^14^C]-citrate over a 4-hr time course for both normal and hepatoma 3924A was clearly established. In addition to [^14^C]-citrate, we also used [^14^C]-acetate as substrate for the PMS, bypassing the ATP-citrate lyase conversion of citrate to acetyl-CoA, the immediate carbon source for cholesterogenesis. Both substrates yielded near identical results (Figure 6A). We noted, as well, that the tumor PMS exhibited a more than 9-fold greater ATP-citrate lyase activity than the normal system (Coleman and Sepp-Lorenzino, 1990), an observation later substantiated by others (Zaidi et al., 2012; Wang et al., 2017; Figure 6B).

Data reduction on a per-cell basis indicated that the tumor PMS had the capacity to synthesize cholesterol from either citrate or acetate more than 9-fold faster than did normal PMS. When normal liver PMS was supplied with [^14^C]-mevalonate rather than [^14^C]-acetate, the rate of cholesterol synthesis increased by 6-fold, confirming that the PMS from normal tissue retains the rate-limiting site for cholesterogenesis (i.e., HMGR) between acetate and mevalonate. Alternatively, the tumor PMS showed no difference in the 9-fold accelerated rate of cholesterol synthesis when fueled by either acetate or mevalonate, indicating that with the tumor system, regardless of whether the initial cholesterol synthesis substrate was supplied before (acetate) or after (mevalonate) the HMGR rate-limiting step, carbon flux was unchanged.

These results support the carbon flux patterns obtained with both isolated mitochondria, as well as from tissue slice incubations, with normal vs. hepatoma preparations described in subsections “The Road to Mitochondrial Membrane Cholesterol Enrichment,” “Cholesterol-Enriched, Isolated Hepatoma Mitochondria Preferentially Export Citrate,” and “Tracking Carbon Flux With Liver vs. Hepatoma Tissue *ex vivo*: What Happens to Pyruvate-Derived Citrate?” above. Moreover, comparisons of steady-state concentrations of some early cholesterogenesis intermediates (e.g., acetate, acetoacetate, hydroxymethylglutarate, and mevalonate) dramatically mirrored the differences in carbon flux between normal and hepatoma 3924A PMS systems. Normal liver PMS displayed high steady-state levels of HMG-CoA and relatively low amounts of mevalonate. The tumor PMS, in contrast, revealed the reverse pattern (for details, see Azrolan and Coleman, 1989; Coleman and Sepp-Lorenzino, 1990).

HMGR immunopreciptation analyses on isolated microsomal fractions from the PMS of equivalent cell numbers of normal ACI rat liver and Morris hepatoma 3924A demonstrated an approximately 10-fold greater amount of HMGR protein in the microsomal fraction derived from the hepatoma vs. normal liver (Azrolan and Coleman, 1989).

Thus, for the first time, this result strongly suggested that the dramatically enhanced carbon flux from pre-mevalonate intermediates through the rate-controlling step of cholesterogenesis, may be mostly due to the considerably greater accumulation of HMGR in the hepatoma 3924A tumor cell. Of course, this could imply, minimally, that in the time-line of transformation from normal liver hepatocyte to hepatoma, a mutation affiliated with the regulation of either HMGR gene over-expression, or the enzyme’s degradation, had occurred. With respect to cancer, overexpression of HMGR by activation of the gene for the sterol regulatory element binding protein (SREBP) is not fully understood (Brown and Goldstein, 1980; Porstmann et al., 2005; Goldstein et al., 2006), but a close interrelationship between SREBP and cancer’s clinical hallmarks has been described (Bao et al., 2016). As well, ubiquitination and proteasome degradation has received serious attention as a mechanism of feedback regulation of the enzyme (Johnson and DeBose-Boyd, 2018).

### Resurrecting the Truncated Krebs/TCA Cycle: Correlation With the Warburg Effect

#### Mitochondrial Carbon-Flux Traffic Detours in Tumors

**FIGURE 7:**
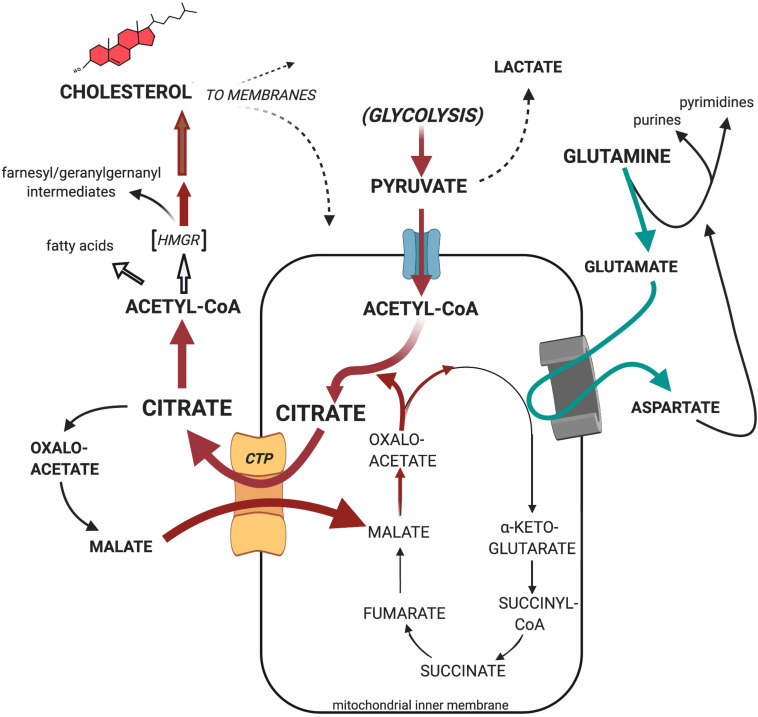
Proposed substrate carbon flow through the truncated Krebs/TCA cycle of tumor mitochondria. Cholesterol-rich tumor mitochondria preferentially export pyruvate-generated citrate as the primary source of cytosolic acetyl-CoA to support the tumor’s enhanced cholesterogenesis, rather than utilize it as substrate for oxidative phosphorylation. Nevertheless, cholesterol-rich tumor mitochondria efficiently engage mitochondrial respiration when fueled, for example, with glutamate together with the malate re-entering in exchange for exiting citrate on the CTP (see text for further details).

Evidence, based on the long-established and repeatedly affirmed observation that tumor mitochondria possess higher membrane cholesterol levels, indicates that as a consequence of such altered membrane lipid composition, the functional behavior of tumor mitochondria can indeed be considered aberrant, as implied by Warburg. Moreover, the fundamental cause of tumor mitochondrial enrichment with cholesterol may be the concomitant loss of regulation of cholesterogenesis at the pathways’s HMGR locus, together with the multifold increase in HMGR per cell, at least as observed with experimental hepatomas. The carbon flux “pull” toward cholesterogenesis may constitute a basis for correlating the re-programmed Krebs/TCA cycle pattern observed in tumors. Our laboratory’s sequential studies on experimental animal hepatomas demonstrated, repeatedly, that a primary and critical metabolic response of cholesterol-enriched mitochondria—whether of tumor origin or artificially achieved via exogenous loading—is the dramatic preferential export of pyruvate-supplied, intramitochondrially generated citrate to the cytosol, where it serves to supply acetyl-CoA, the essential precursor substrate for cholesterol and lipid anabolism. Finally, and most significantly, the effect of depriving citrate from participating in the Krebs/TCA cycle can be definitively circumvented, or at least drastically diminished, by substituting alternative Krebs/TCA cycle intermediates (e.g., glutamate) to fuel respiration-linked oxidative phosphorylation. Such re-routing of citrate occurs independent of the presence of O_2_. Collectively, these observations constitute a strong basis for the proposed continuously operating, “truncated” or abbreviated Krebs/TCA cycle, whereby faster-growing tumors export the bulk of the citrate from the mitochondria to the cytosol, thereby reinforcing Warburg’s impaired mitochondrial respiratory profile. Moreover, as would be inferred as a consequence of the Warburg effect, enhanced glycolysis, in concert with deregulated cholesterogenesis, might realize both an overproduction of cholesterol and provide for ATP production, with the export of citrate featured as a key element of the tumorigenic process (Figure 7).

A striking manifestation of respiratory substrate re-routing that evolves from the proposed truncated Krebs/TCA cycle by preferential citrate export from tumor mitochondria, is the well-established elevation in mitochondrial glutaminolysis activity in cancer, and the correlative utilization of resulting glutamate as Krebs/TCA cycle participant upon its conversion to α-ketoglutarate by glutamate dehydrogenase or, as shown in Figure 7, aspartate aminotransferase (Reitzer et al., 1979; Frigerio et al., 2008; Erickson and Cerione, 2010; Wise et al., 2011; Matés et al., 2019). And, as Figure 7 illustrates, glutamine is known to serve multiple key roles in cellular metabolomics, both in normal and pathologic tissues (Hensley et al., 2013; Cluntun et al., 2017). Such remodeling of the tumor’s respiratory substrate utilization effectively *supplements* the tumor’s enhanced glycolitic profile, rather than being supplanted by it (DeBerardinis and Chandel, 2016). It underscores the operational malleability of tumor bioenergetics (i.e., oxidative phosphorylation) concomitant with the anabolic diversion of citrate toward the latter’s incorporation into the tumor’s deregulated cholesterogenesis *de novo* as depicted in Figure 7, and documented in this Review (section “Mitochondrial Metabolism Is Anomalous in Tumors,” subsection “Tracking Carbon Flux With Liver vs. Hepatoma Tissue *ex vivo*: What Happens to Pyruvate-Derived Citrate?”).

SF188 glioblastoma cultures, exhibiting a pronounced Warburg effect, were examined in an elegant and comprehensive study by DeBerardinis et al. (2007). ^13^C-NMR was employed with ^13^C-labeled glucose, thus allowing for real-time, simultaneous analysis of multiple metabolic pathway carbon flux patterns in these cells. Their data dramatically revealed several important features of carbon flux in these gliomas. They found the major route of carbon entry into the Krebs/TCA cycle for ^13^C-glucose-derived pyruvate-to-acetyl-CoA conversion is through mitochondrial pyruvate dehydrogenase. Further, they confirmed the anapleurotic utilization of glutamate in the Krebs/TCA cycle, and, most importantly, they substantiated that 60% of the ^13^C-glucose tracer was incorporated into newly synthesized lipids, necessitating the efficient mitochondrial export of ^13^C-citrate followed by cytosolic conversion to ^13^C-acetyl-CoA. This study also confirmed, as had our own hepatoma work (Parlo and Coleman, 1986), that despite the tumor’s distinct Warburg effect profile, the Krebs/TCA cycle performs unimpaired, although by way of an amended carbon flux.

With the preferential export of tumor mitochondrial citrate via the CTP resulting in a diminished availability of citrate to directly support TCA cycle-linked respiration, and the elevated conversion to cytosolic acetyl-CoA as precursor for deregulated cholesterogenesis, one begins to discern the outlines of a more general, over-arching metabolic pattern for tumor growth and proliferation.

To support the main tenet of the Warburg effect and the unrestrained cell proliferation of tumors—i.e., an increased dependence on aerobic glycolysis with diminished reliance on O_2_-linked oxidative phosphorylation—we postulate an advantageous and, we emphasize, continually maintained carbon flux pattern; one that expands and supplements the metabolic focus of analysis beyond the prevalent mitochondria-centered considerations. Such a highlighted tumor-specific, more broadly viewed metabolic segment, would consist of the following sequence of events:

1. Cytosolic pyruvate enters mitochondria long enough to generate citrate, which no longer will become engaged in the TCA cycle;
2. but, upon accelerated exit to the cytosol, instead becomes the critical substrate pool precursor for de-regulated cholesterogenesis;
3. whose diverse mevalonate-derived isoprenyl intermediates serve as imperative triggers for DNA synthesis.

#### Glycolytic Response to Accelerated Cholesterogenesis in Tumors

Continuous, accelerated carbon flux from extramitochondrial pyruvate to cholesterol requires a coordinated and compensatory higher glycolytic activity. This reorganized carbon flow necessitates an escalated uptake of glucose into tumors, leading, ultimately, to an abnormal cholesterol enrichment of tumor cell membranes. The tumor’s increased dependence on higher glycolytic carbon flux, corresponding with the Warburg effect requirements, takes advantage of many levels of metabolic signaling that shift emphasis toward anabolic profiles in the cytosol in order to prepare cells for impending proliferation. Here, the effect of enhanced mitochodrially exported cytosolic citrate is not to help regulate glycolysis by allosteric control of phosphofructokinase, as proposed in normal cells, but to serve as a constantly refreshed source of cytosolic precursor substrate for cholesterogenesis in tumors.

The classic regulation of glucose uptake and ensuing glycolysis has been reviewed in detail elsewhere (Cox and Nelson, 2005; Voet and Voet, 2011). However, select aspects of the exceedingly complicated and varied regulatory signals controlling glucose metabolism are abbreviated here to support peripheral evidence for the remodeling of glucose-fed tumor cell carbon flux. Generally speaking, considerable evidence indicates glycolysis enzymes are upregulated in tumors (Cairns et al., 2011).

Foremost among controlling elements is the dominant role in directing tumor cell glucose utilization via protein phosphorylations supervised by the phosphoinositide 3-kinase (PI3K)/serine-threonine-specific protein kinase (Akt) —PI3K/Akt cascade—along with another serine-threonine kinase called “mammalian-target-of-rapamycin” (mTor) (Vander Heiden et al., 2010; Hanahan and Weinberg, 2011; Fruman and Rommel, 2014). This family of signaling proteins has been shown to be constitutively amplified in tumors (Elstrom et al., 2004; Fruman and Rommel, 2014).

Upon activation of receptor tyrosine kinases on the plasma membrane cell surface, the PI3K/Akt pathway signals several glycolysis-linked components that stimulate glycolysis carbon flux. PI3K/Akt activity: (1) causes increased expression, and thus activity, of the cell’s plasma membrane-embedded glucose transporter GLUT1, yielding increased glucose uptake; (2) promotes hexokinase (HK-II) mitochondrial-outer-membrane VDAC association, facilitating creation of glucose-6-phosphate; and (3) indirectly stimulates phosphofructokinase (PFK-I) activity to generate increased fructose 1,6,-fructose-*bis*-phosphate, the rate-limiting reaction of glycolysis. All of these effects act synergistically to stimulate enhanced glycolysis in tumors independent of the presence of O_2_, consonant with the Warburg effect.

Insofar as regulation of glycolytic carbon flux, particularly with respect to tumors, cytosolic citrate is a commonly prescribed allosteric inhibitor of phosphofructokinase activity (Sola et al., 1994; Usenik and Legiša, 2010). But in the case of the tumor’s deregulated and dramatically accelerated cholesterogenesis, where the steady-state pool of citrate is kept from accumulating as it continuously supplies the acetyl-CoA for lipid anabolism, it is doubtful if citrate plays a significant role in this regard.

When the glycolytic rate becomes excessive and less responsive to multi-levels of control, as in rapidly proliferating tumors, numerous metabolic ramifications become apparent which alter the cell’s homeostasis. Thus, we can contemplate the fate of the end product of the pathway, pyruvate, positioned at a crossroad of further metabolism. While in the cytosol, pyruvate may be either reduced to lactate by lactate dehydrogenase (LDH), or become transported into mitochondria on the inner membrane pyruvate carrier (MPC) (Bender and Martinou, 2016; Rauckhorst and Taylor, 2016), where it will become engaged in TCA cycle mechanics. Not surprisingly, LDH over-expression also has been reported in tumors (Cui et al., 2014; Mishra and Banerjee, 2019). If the rate of glycolytic flux in tumors outpaces or overburdens the capacity of mitochondrial uptake of pyruvate, lactate production will predominate, allowing partial restoration of the cytosolic pool of NAD^+^ that was required during operation of glycolysis.

On the other hand, cytosolic lactate accumulation can obtain when the tumor’s environment compels its metabolic machinery to respond flexibly by means of divergent pathways. An example is the processing of malate either by the cytosolic NADP^+^-requiring malic enzyme, which oxidatively decarboxylates malate to regenerate pyruvate, and concomitantly provides NADPH used in lipid biosynthesis, or by the NAD^+^-requiring mitochondrial malic enzyme variant that participates in the alternative fueling of the truncated Krebs/TCA cycle by glutaminolysis (Vacanti et al., 2014; Yang et al., 2014).

Yet, tumor mitochondrial pyruvate dehydrogenase (PDH) is not only active, but can provide for substantial glucose carbon flux into lipogenic acetyl-CoA (Holleran et al., 1997). Such results indicate that despite a seeming “competition” between cytosolic LDH and mitochondrial MPC for disposal of glycolysis-derived pyruvate carbons, a considerable flow of pyruvate carbons into the TCA-cycle to generate citrate via acetyl-CoA can and does occur. And, as was noted previously via the extensive ^13^C-NMR analysis in glioblastoma cells (DeBerardinis et al., 2007; Yang et al., 2014), the carbon flux of ^13^C-glucose-derived pyruvate-to-acetyl-CoA conversion not only confirmed unhindered participation of pyruvate dehydrogenase, but that respiratory-linked oxidation of the citrate generated by the TCA cycle in these tumors was limited by its efflux from mitochondria in order to support lipid synthesis. Such ^13^C-flux routing of metabolism that demonstrated diminished mitochondrial citrate-fueled oxidation as a consequence of preferential lipid biosynthesis, both supports and confirms the data obtained with both cholesterol-enriched hepatoma mitochondria as well as with normal mitochondria exogenously enriched with cholesterol (Parlo and Coleman, 1984), described above (section “Mitochondrial Metabolism Is Anomalous in Tumors,” subsection “Cholesterol-Enriched, Isolated Hepatoma Mitochondria Preferentially Export Citrate”). Moreover, experimentally manufactured CTP-deficient lung cancer cells were found to display dramatically re-programmed Krebs/TCA cycle metabolomics relative to their normal cell counterparts (Jiang et al., 2017), including enhanced glycolysis and lactate production, mirroring a principal Warburg effect motif.

#### Cell Cycle Consequences of Mitochondrially Effluxed Citrate—Cell Proliferation Requires Mevalonate-Derived Prenylated Protein Signaling and Adequate Membrane Cholesterol

Carbon flux through the cholesterogenesis pathway, long known as requisite for DNA synthesis and cell proliferation (Chen et al., 1975; Quesney-Huneeus et al., 1983; Fairbanks et al., 1984; Siperstein, 1984) is also recognized to be temporally coordinated with discrete intervals of the four sequential cell cycle phases that occur between successive mitoses (G_0_, G_1_, S, and G_2_) (Sánchez-Martín et al., 2007). With synchronized cells in culture, carbon flux into *de novo* cholesterol is low during early G_1_, increases rapidly to peak midway into G_1_, and declines at the G_1_/S interface. Abundant research data indicate that specific inhibition of cholesterogenesis at the HMGR locus prevents cells from progressing through the G_1_/S boundary into DNA synthesis and cell division, a blockage that was repeatedly shown to be circumvented by addition of mevalonate, but not by the exogenous supplementation of cholesterol to the cultures (reviewed in Coleman et al., 1997). A more recent study, however, presented a unique case of contrasting results. Singh et al. (2013), observed that blocking HMGR activity in F111 fibroblast cultures with either a statin drug (which the authors termed a “proximal” pathway inhibitor), or by inhibiting the last-step conversion of desmosterol to cholesterol with triparanol (considered a “distal” pathway inhibitor) arrested the cell cycle in G_1_, prior to S phase (DNA synthesis), stopping cell cycle progression and mitosis. However, supplying serum cholesterol to these cells appeared to be the *only* means of reversing the cell cycle blockage effected by either “proximal” or “distal” cholesterogenic inhibitors. Such disparate experimental outcomes highlight our insufficient molecular understanding about the consequences to cell proliferation by post-mevalonate intermediates, despite some of the more salient discoveries, briefly summarized here, on mevalonate-derived polyisoprenoids produced *en route* to the creation of cholesterol.

Undeniably, among the more exciting research discoveries relevant to cholesterogenesis was the discovery of mevalonate-derived, prenylated proteins. The lengthening isoprenoid intermediates generated by the mevalonate-to-cholesterol pathway features a number of diversionary branches off the main cholesterogenic route. These branch points, yielding polyisoprenyl side chains, have been found to covalently modify an assortment of proteins involved in signaling cell proliferation. Among the most influential of these mevalonate-generated isoprenyl intermediates are the farnesyl- (C_15_) and geranylgeranly- (C_20_) moieties (Casey, 1992, 1995; Sebti, 2005; Wang and Casey, 2016), catalyzed by farnesyltransferase (or geranylgeranyltransferase) enzymes. These enzymes effect covalent modification of the Ras superfamily of plasma membrane-associated G-proteins, as well as various cytoskeletal Rho and nuclear lamin proteins, along with other potential cell cycle signaling species which influence the activity of the cyclin family of proteins that regulates passage through the cell cycle (Rahman and Kipreos, 2010). Considerable attention continues to be focused on elucidating potential roles of prenylated proteins as signaling elements in cell proliferation because, among other observations, specific inhibition of the farnesyl protein transferase enzyme elicits upregulation of a protein (p21^*Waf1/Cip1*^) involved in cyclin control of cell cycle progression (Sepp-Lorenzino et al., 1991; Sepp-Lorenzino and Rosen, 1998; Tamanoi et al., 2001).

Such mevalonate pathway details, although vitally important, distract scrutiny of the accelerated, continuous, citrate-supplied, carbon flux through cholesterognesis, and the resulting membrane cholesterol enrichment that helps define the Warburg effect phenotype of tumors. Meaningful to the theme of this review was a study that demonstrated the tight coupling between the rate of mevalonate availability and the rate of protein prenylation in cultured murine erythroleukemia cells (Repko and Maltese, 1989). A principal result of these experiments showed that inhibition of protein synthesis with cycloheximide almost immediately abolished [^3^H]-mevalonate incorporation into prenylated proteins. But inhibition of HMGR by a statin diminished [^3^H]-mevalonate incorporation into susceptible proteins over a slower time-course, suggesting two correlated events: (1) protein prenylation from the available pool of mevalonate occurs very rapidly upon synthesis of susceptible proteins (including, of course, the responsible prenyltransferase enzymes); (2) the rate of mevalonate manufacture—i.e., the carbon flux through cholesterogensis—determines the pool size, and thus, the availability of isoprenyl intermediates for covalent protein modification. This conclusion reminds us that in synchronized cell cultures, the inhibition of HMGR during early G_1_ obliterates manufacture of not only mevalonate, and therefore of cholesterol (whose synthesis peaks in mid-G_1_ and declines at the G_1_/S boundary) but prevents DNA synthesis and cell division. To restart cell growth at this synchronized stage, both mevalonate and cholesterol are required. Yet, if HMGR is inhibited after the G_1_-peak cholesterol synthesis occurs, DNA synthesis is restored merely by mevalonate addition (Sinensky and Logel, 1985; Langan and Volpe, 1987). This result is consonant with the findings of Repko and Maltese (1989), and implies the cholesterol-independent requirement of mevalonate-derived prenylated proteins as feasible signaling triggers for DNA replication.

#### BTC Inhibition of CTP Arrests (Reversibly) Cholesterol Synthesis and DNA Replication in Synchronized Tumor Cells

After cholesterol synthesis has peaked in mid-G_1_, the synthesis of new DNA in preparation for cell division requires a constantly re-supplied pool of mevalonate. How might the effects on DNA synthesis be explored if the supply of mevalonate substrate precursors, such as acetyl-CoA (or *its* cytosolic precursor, citrate), were limited? As described previously in this review, our studies with *ex vivo* liver and hepatoma incubations and isolated liver and hepatoma mitochondria, utilized the CTP inhibitor BTC to encourage and reinforce the proposal of a truncated Krebs/TCA cycle (Figure 7), a paradigm for the Warburg effect in cholesterol-enriched tumor mitochondrial membranes.

As detailed, BTC inhibition of the CTP, in both hepatoma and exogenously cholesterol-enriched normal liver mitochondria, re-established the participation of mitochondrial citrate as substrate for oxidative phosphorylation (Figure 5). Simultaneously, BTC blockage of CTP eliminates the continual cytosolic citrate re-supply via mitochondrial exchange export for malate, and thus would starve the cell of mevalonate carbons required for cholesterogenesis and ensuing DNA replication.

**FIGURE 8:**
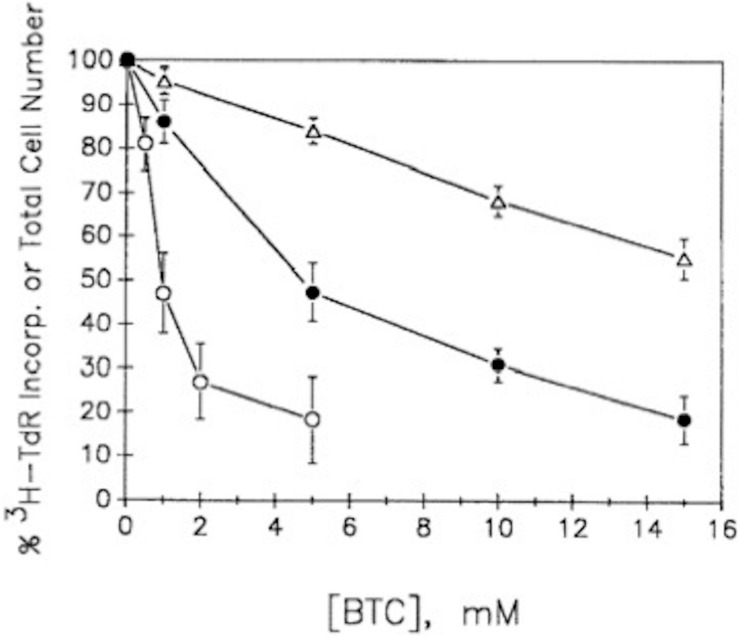
BTC inhibits cell growth and [^3^H]TdR incorporation into DNA. Exponentially growing unsynchronized or double-thymidine-block-generated G_1_/S synchronized 70Z/3 murine lymphoma cultures were incubated with [^3^H]TdR together with the BTC concentrations indicated (see Rao and Coleman, 1989, for experimental details). Synchronized cultures were found to incorporate maximal [^3^H]TdR about 3 h after release from the thymidine blockade. Data show means ± SEM for three experiments (12 replicate samples/experiment). △, % of total number of unsynchronized cells; •, % of [^3^H]TdR incorporation into unsynchronized cells; ○, % of [^3^H]TdR incorporation into G_1_/S synchronized cells (adapted with permission, Rao and Coleman, 1989).

**FIGURE 9:**
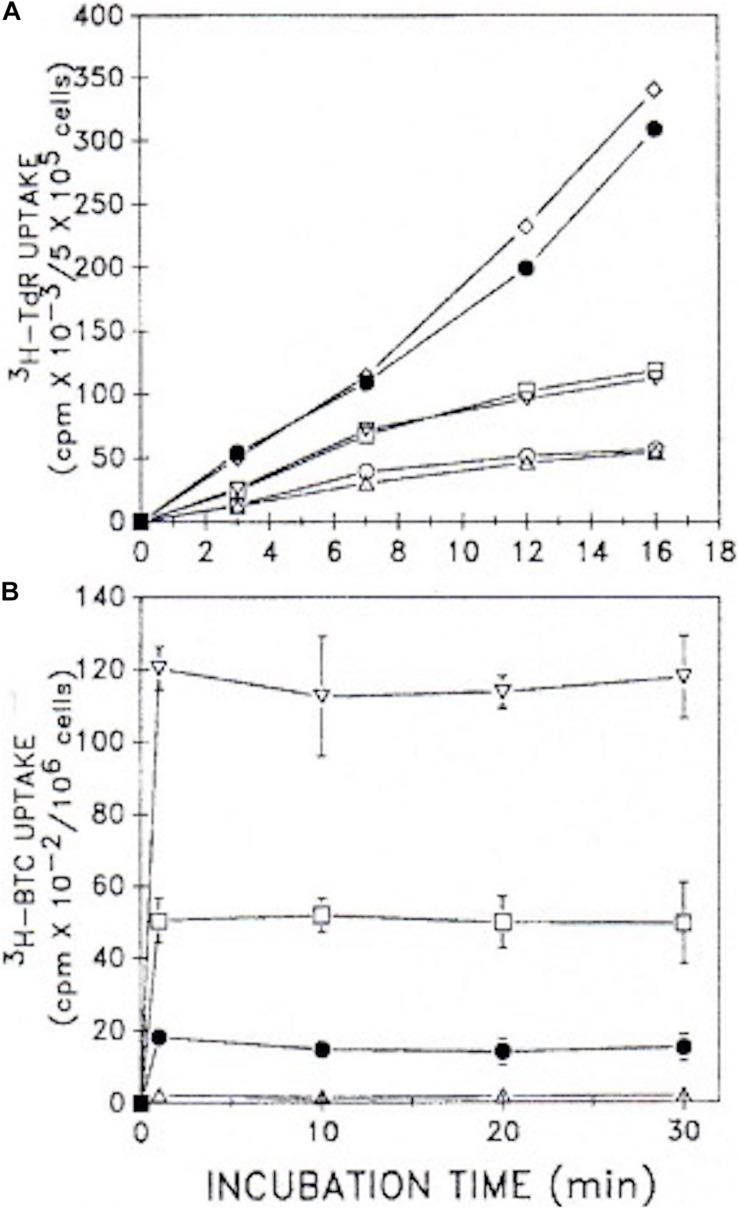
Uptake kinetics of [^3^H]TdR and [^3^H]BTC into cells. **(A)** [^3^H]TdR uptake kinetics were assessed in unsynchronized 70Z/3 murine lymphoma cultures (5 × 10^5^ cells/ml) *plus* and *minus* 10 mM BTC. Cells were incubated for the times indicated with varying [^3^H]TdR concentrations. Mean values of two experiments performed in duplicate are shown. △, □, •, minus BTC; and ○, ▽, ◇, plus 10 mM BTC, with 1, 3, or 6 μCi [^3^H]TdR under both conditions, respectively. **(B)** [5-^3^H]BTC uptake kinetics were assessed in unsynchronized cultures (10^6^ cells/ml) over the time course shown. Increasing concentrations of BTC (0.1–10 mM) together with [5-^3^H]BTC were employed under a constant specific activity of 1 μCi/0.1 mM. Two experiments performed in duplicate (±SD) are given. △, with 0.1 mM BTC; •, with 1 mM BTC; □ with 5 mM BTC; ▽, with 10 mM BTC (adapted with permission, Rao and Coleman, 1989).

Our observations (Rao and Coleman, 1989) with both G_0_/G_1_-synchronized, as well as unsynchronized, proliferating murine lymphoma (70Z/3) cultures (≤10^6^ cells/ml), demonstrated that BTC (between 1 and 10 mM) not only inhibited [^14^C]-pyruvate incorporation into cholesterol, but concomitantly inhibited [^3^H]-thymidine incorporation into DNA, thereby arresting cell proliferation (Figure 8). We were impressed that BTC, despite its aromaticity and sparing solubility in the pH 7 environment of cellular homeostasis, very rapidly transited the plasma membrane, dispersed within the cell, and, within 1 min, manifested its metabolic effects on carbon flux and DNA synthesis (Figure 9). Furthermore (and surprisingly), these BTC-induced metabolic inhibitions proved completely reversible upon washing the cells free of BTC, without exhibiting deleterious effects on cell viability. BTC, thus, appeared non-cytotoxic, at least with this tumor cell system.

We confirmed that citrate’s well-known metal chelating ability (Mg^2+^, Ca^2+^) is shared by BTC. Attempts to even partially overcome the BTC inhibition of DNA replication by the addition of 0.5 mM mevalonate to the culture required inclusion of the plasma membrane cation ionophore A23187 to the incubation. This permitted cytosolic Ca^2+^/Mg^2+^ to become replenished from the culture medium (∼0.5 mM), compensating, partially, for the chelating effects of BTC, and illuminating the mevalonate pathway’s well-established promotion of DNA replication (Rao and Coleman, 1989).

The BTC-implemented metabolic shift of pyruvate-supplied carbons, and its effect on DNA synthesis, proved to unconditionally require the cellular cytosolic environment (mitochondria, ER, etc.). Experiments with isolated nuclear preparations from the 70Z/3 murine lymphoma affirmed that DNA synthesis was not significantly inhibited in BTC-treated, mevalonate-supplied incubations, but could be completely restored merely by addition of 5 mM Mg^2+^. In these studies adequate levels of serum cholesterol were available to the cultures, signifying that the documented BTC effects on DNA synthesis were not due to cholesterol or fatty acid starvation.

Analysis of further experimental permutations with this tumor system (Rao and Coleman, 1989) gave authority to the following conclusions: (1) Inhibition of both pyruvate-fueled cholesterol and DNA syntheses are direct intracellular metabolic responses to BTC, not inhibitor uptake issues; (2) BTC inhibition proved non-toxic, allowing complete recovery of DNA replication and cell viability after removal; (3) Blocking the mitochondrial CTP at the end of G_1_, limiting the supply of cytosolic citrate and thus acetyl-CoA, and thereby ultimately mevalonate, arrests further progression through S-phase, abruptly halting cell proliferation.

In terms of the generally protracted time-line of tumor cell growth, therefore, accumulated data accentuate these tightly linked metabolic hallmarks of uncontrolled cell proliferation: deregulation of cholesterogenesis at the HMGR metabolic locus; mitochondrial membrane enrichment with cholesterol; preferential export of pyruvate-generated (glycolysis-generated) citrate from cholesterol-enriched mitochondria; and the requirement of a continual supply of mevalonate for ensuing DNA synthesis and cell replication. Collectively, these metabolomic profiles coincide to help endorse our proposed “truncated Krebs/TCA cycle” model (Figure 7).

### Deregulated Cholesterogenesis Promotes Vesicle Exfoliation From Cholesterol-Rich Plasma Membrane Lipid Raft Domains

Nearly a decade before the introduction of the collective term “exosomes” to characterize exfoliated vesicles from normal and tumor cells (Trams et al., 1981), research demonstrated the shedding of vesicular plasma membrane fragments into the cell-free ascites fluid from various murine leukemias and lymphomas, as well as in the sera and pleural effusions of leukemia patients (Grohsman and Nowotny, 1972; Raz et al., 1978; van Blitterswijk et al., 1979). Further investigations restricted the exosome nomenclature to vesicles of endosomal derivation based on the protein components within their membranes, or by the cargo enclosed within them, compared with extracellular microvesicles (sometimes called “ectosomes”) shed from the plasma membranes of healthy, growing cells, whether of normal or pathological origin (Raposo and Stoorvogel, 2013). There remains nomenclature confusion over the biogenesis of shed membrane vesicles, which are referred to merely as extracellular vesicles (EV) (Kato et al., 2020).

Despite recent decades employing sophisticated membrane cholesterol imaging and other analytical techniques that have elucidated the roles and evolution of shed vesicles (whether exosomes or ectosomes) in intercellular trafficking, and their potential involvement in immune surveillance (Raposo and Stoorvogel, 2013; Record et al., 2014; Litvinov et al., 2018; Kato et al., 2020), cholesterol’s organization in the cholesterol-rich lipid raft microdomains of the plasma membrane remains poorly understood (Schroeder et al., 2010). Evidence shows the distribution of cholesterol between lipid bilayer leaflets in plasma membranes is not equal, with the cytosol-facing leaflet cholesterol-enriched by as much as 40 mol% of the lipids in that leaflet (Mondal et al., 2009). The greater lateral “rigidity” of this inner-facing vs. the exterior-facing bilayer leaflet has biologic (Pike, 2003) as well as physical manifestations (Vámosi et al., 2006). An early theoretical model, potentially applicable to membrane vesicle exfoliation, was based on free-energy calculations of the plasma membrane’s inner leaflet surface area expansion, due to asymmetric (enhanced) cholesterol incorporation (Luke and Kaplan, 1979). Mathematical modeling predicts an outwardly growing spherical distortion of the bilayer, developing into a dumbbell-shaped, narrow-necked structure that eventually pinches off, releasing the cholesterol-rich lipid spheroid to the exterior milieu in an attempt to re-establish the pre-enrichment bilayer lipid composition and surface area. Other models have been proposed (Khatibzadeh et al., 2013). Whether or not such modeling for microvesicle formation is applicable to the shedding of cholesterol-rich vesicles from tumors, convincing evidence exists that cholesterol-rich lipid raft domains play unambiguous, although incompletely understood, roles in the variety of modes that characterize cancer’s accelerated pathological and immunosurveillance situations (Pfrieger and Vitale, 2018; Vu et al., 2019).

Mitochondrial membrane cholesterol enrichment in the graded growth series of Morris hepatomas 16 and 3924A (Parlo and Coleman, 1984, 1986) prompted initiation of further study of possible correlations between enhanced, deregulated cholesterol synthesis and the augmentation in membrane cholesterol content of tumor plasma membranes, as well as that of exfoliated microvesicles isolated from the cell-free ascites fluid (CFAF) of a chemically induced murine hepatoma (MH-129) carried in C3H mice. The results revealed both far-reaching and corroborative data germane to the theme of this review, linking the Warburg effect, the proposed truncated Krebs/TCA cycle, and the continuing carbon flux from tumor mitochondria to newly synthesized cholesterol (Puma, 1982).

Although no longer the case (Fonsato et al., 2012), at the time these studies were undertaken (Puma, 1982) little information existed about vesicle exfoliation from hepatomas. The MH129 ascites hepatoma used in these early investigations was readily grown in culture, C3H mouse liver served as the normal (control) membrane source, and, most importantly, MH129 was known to have no viral etiology to complicate the biologic derivation of the vesicles shed into the CFAF. The latter fact was confirmed via transmission electron microscopy of the isolated membrane fraction, which revealed no evidence of ER contamination in the CFAF, and disclosed dimensional aspects of the vesicles consistent with contemporary (van Blitterswijk et al., 1979) or current (Raposo and Stoorvogel, 2013) isolated membrane microvesicle preparations. Multiple marker enzyme assays confirmed the identity of the isolated plasma and shed microvesicle membranes.

These studies (Puma, 1982) showed that the MH129 tumor cell homogenate contained nearly 30% more cholesterol compared with that of normal C3H mouse liver. Microvesicles shed from these cholesterol-enriched hepatoma cells, recovered from the CFAF, demonstrated a 77% greater cholesterol content than the plasma membranes isolated from the MH129 tumor itself, and possessed very substantial increases in the activities of the plasma membrane marker enzymes 5′-nucleotidase and Na^+^/K^+^-ATPase. Significantly, when the ascites culture was fed 54 μg mevalonate/10^6^ cells for 3 h, the microvesicles exfoliated into the CFAF were cholesterol enriched 81% more than the shed vesicles from cultures *minus* added mevalonate. The data emphasize three important features that uphold the preferential carbon-flow concept of mitochondrially exported citrate that feeds deregulated, enhanced cholesterogenesis in tumors: (1) exfoliated vesicles contain higher amounts of cholesterol than the tumor plasma membranes from which they were shed; (2) supplying mevalonate to ascites tumor cultures dramatically yields an even further increase in the amount of cholesterol in the exfoliated microvesicles; and (3) the process of cholesterol-enriched microvesicle shedding in these tumors not only occurs both *in vivo* and *in vitro*, but is continuous and, *in vitro*, rapid (within 3 h), highlighting the tumor’s accelerated carbon flux through cholesterol synthesis anabolism.

### Contemplating Coordinated Operation Between CTP/ *SLC25A1* and PMCT/*SLC13A5* for Citrate-Sustained Control of Glycolysis and Cholesterogenesis in Tumors

Management of the supply chain of such a critical anabolic fuel as cytosolic citrate must be flexible enough to compensate for its deficit, if biosynthetic demand for citrate’s carbons outstrips availability. Due to a cancer cell’s heterogenous stromal tissue environment *in vivo* [prostate cancer is a prime example—(Tolkach and Kristiansen, 2018)] and the fact that tumors derived from different tissues present varying metabolomic profiles, including highly varying glycolytic and mitochondrial respiratory rates, the cytosolic demand for biosynthetically employed citrate would be expected to vary.

The two well-known means of conveying citrate into the cytosolic pool are the mitochondrial citrate transporter CTP/*SLC25A1* and the more recently established plasma membrane Na^+^-dependent citrate transporter PMCT/*SLC13A5*. Both membrane transporters are considered prime focal points for the development of cancer chemotherapeutic drug development (Catalina-Rodriguez et al., 2012; Huard et al., 2015; Mycielska et al., 2019). It is of significant interest to consider if and how both transporters might coordinate the steady-state level of cytosolic citrate, and thereby serve as cooperative means of adjusting the tumor’s carbon flux through our proposed truncated Krebs/TCA cycle, accelerated cholesterogenesis, and the resulting enhanced glycolysis that together help characterize the Warburg effect profile.

Of the more than 50 mitochondrial membrane substrate/ion transporters of the SLC25 superfamily that have been identified and characterized to date (for reviews see: Kaplan, 2001; Palmieri and Monné, 2016), Kaplan’s laboratory (Remani et al., 2008; Aluvila et al., 2010; Sun et al., 2010; Nie, 2017) has provided significantly detailed information on the structure, substrate transport mechanism and analysis of inhibition by BTC and other potential inhibitors of the citrate-malate CTP carrier, as well as on the PMCT membrane transporter. PMCT is the more recently characterized plasma membrane citrate transporter variant in humans and has garnered considerable attention since it serves as an additional (and, so far only) channel for the influx of citrate into the cytosol from the extracellular space (Mycielska et al., 2009). A potent synthetic dicarboxylate inhibitor of the PMCT has been described (Huard et al., 2015; Pajor et al., 2016), and surprisingly, gluconate, an abundant natural oxidation product of glucose, is a competitive irreversible inhibitor of the PMCT (Mycielska et al., 2019). The inhibition of both plasma membrane and mitochondrial citrate transporters in experimental hepatomas (by moieties the authors merely designated CTP_*i*_ and PMCT_*i*_) was reported to dramatically inhibit lipogenesis, enhance apoptosis and induce cell cycle arrest (Poolsri et al., 2018). In studies comparing malignant (PC-3M) and benign epithelial prostate cells, as well as other tumor and normal cells, the proliferating cancers were consistently found to take up greater amounts of citrate than their normal counterparts via the PMCT (Mycielska et al., 2018). Significantly, prostate tumor cells have been shown to possess enriched levels of cholesterol (Murtola et al., 2012).

CTP/*SLC25A1* and PMCT*/SLC13A5* comprise the only known membrane-partitioned, compartmentally separated supply routes for adjusting homeostatic levels of cytosolic citrate in humans. Despite obvious reprogramming of metabolism that establishes the tumor’s deregulated cholesterogenesis which encourages its cell proliferation phenotype, and unlike the case with the CTP of hepatoma mitochondria documented previously in this review, we are not aware of studies that focus on correlating effects between the specific cholesterol level of tumor plasma membranes and the activity of the PMCT. Yet, sophisticated techniques are now available (Liu et al., 2017; Hu et al., 2019) that allow the positional sensing of cholesterol within micro domains of the plasma, or the exfoliated vesicle, membrane bilayer. Given a particular tumor’s transformable metabolic adaptability, the cholesterol content of lipid rafts in the lateral bilayer membrane environment of the PMCT might modulate the passage of citrate into or extrusion from the tumor cell, and thereby exert a pleiotropic influence on the manner in which citrate is employed catabolically and anabolically in cancer.

## Summary

This review, a reminder of the prescience of the Warburg effect and its influence on half a century of molecular-level cancer research, has been designed to reignite awareness of the dominant and continuous metabolic carbon flux in tumors whose uncontrolled growth fundamentally depends upon two linearly linked metabolic patterns: (1) the ubiquitously observed loss-of-control of HMGR activity promoting an elevated cholesterol biosynthesis rate, the operation of which is mandatory for DNA synthesis and cell division, and fueled by cytosolic citrate; (2) preferential metabolic routes, involving critical participation of the mitochondrial CTP and, potentially, the plasma membrane PMCT, through which an augmented supply of cytosolic citrate is sustained. Herein, we have enlarged the scope of tumor cell metabolomics by considering not only the mitochondrial bioenergetic carbon flux patterns in tumors and their adherence to the Warburg effect, but by emphasizing the paramount role assumed by cytoplasmic citrate, we have delineated consequences for tumor cell proliferation when accelerated cholesterogenesis prevails. Thus:

1. The molecular basis for the absence or reduced control of citrate-fueled cholesterogenesis in tumors has been traced to a combination of defective feedback control elements and/or proteolytic degradation failures at the rate-controlling HMGR step of the synthetic pathway, in concert with a constitutively elevated expression of the HMGR enzyme. Our own research documented a 10-fold increase in the HMGR protein/cell in a rapidly proliferating heptoma, and a corresponding 9-fold increase in the rate of citrate-fueled cholesterol synthesis, compared with the normal liver system. Notwithstanding the cause, continuously escalated tumor cholesterogenesis requires a commensurately elevated and continuous supply of citrate as its fundamental biosynthetic substrate.
2. Membranes of tumor cell mitochondria, as well as their plasma membranes, have been long known to possess more cholesterol than those of their normal tissue counterparts. *In situ*, the extent of such cholesterol enrichment appears to correlate with the proliferative rate of the particular tumor and the heterogeneity of its tissue environment.
3. Mitochondria from an increasingly graded growth series of rat hepatomas display corresponding enrichment with cholesterol, with directly correlating increased rates of citrate export, and proportionate curtailment of pyruvate- or citrate-fueled O_2_ consumption. Exogenous enrichment of normal liver mitochondria with titratable increased levels of cholesterol mimics this abnormal curtailment of early substrate Krebs/TCA cycle respiration in tumor mitochondria, proportional to the level of cholesterol enrichment. By blocking citrate export, the specific CTP inhibitor BTC completely reverses the decrease in pyruvate- and citrate-fueled respiration associated with aberrant membrane cholesterol enrichment. Yet, under either tumor or exogenously cholesterol-enriched-normal mitochondrial experimental conditions, fueling mitochondrial respiration-linked oxidative phosphorylation with substrates that enter the Krebs/TCA cycle beyond the aconitate hydratase step yields normal rates of O_2_ consumption. Thus, mitochondrial membrane cholesterol enrichment occurring during the progression from normal-homeostatic to tumorigenic-cell proliferative states directly effects increased CTP activity and drives accelerated export of mitochondrial citrate into the cytosol (Section “Mitochondrial Metabolism Is Anomalous in Tumors,” subsection “Cholesterol-Enriched, Isolated Hepatoma Mitochondria Preferentially Export Citrate”).
4. These aberrant tumor respiratory patterns with isolated hepatoma mitochondria are replicated in *ex vivo* hepatoma tissue slice incubations fueled with pyruvate or citrate. Furthermore, following the preferential utilization of either substrate enabled the tracking of the carbon flow either through the tumor’s deregulated cholesterogenesis pathway to cholesterol, or into the CO_2_ end-product of normal tissue respiration. As with the isolated mitochondrial experiments, BTC reverses the pattern by preventing export of mitochondrially generated citrate to the cytosol, thus depriving the cholesterogenesis pathway of its precursor source of cytosolic acetyl-CoA (section “Mitochondrial Metabolism Is Anomalous in Tumors,” subsection “Tracking Carbon Flux With Liver vs. Hepatoma Tissue *ex vivo*: What Happens to Pyruvate-Derived Citrate?”). Considered together, these results are congruent with our proposed “truncated” Krebs/TCA cycle (Figure 7) portraying an altered metabolomic carbon flux that, in concert with an enhanced glycolytic rate, and regardless of the tumor environment’s O_2_ level, satisfies the deduced mitochondrial dysfunction originally proposed by the Warburg effect.

5. The critical importance of supplying cytoplasmic citrate from mitochondria as prerequisite for cell cycle progression is well documented. We demonstrated that BTC, the classic CTP inhibitor, rapidly enters exponentially growing ascites lymphoma cell cultures and arrests DNA synthesis (Figures 8, 9). BTC can transit the tumor cell’s plasma membrane (apparently in both directions) with ease, and its inhibitory effect on CTP is both sensitively expressed and appears non-toxic, since the arrest of DNA synthesis and termination of cell replication is reversible upon BTC’s removal from the culture. Addition of BTC starved the cytosol of mitochondrially exported citrate and inhibited DNA replication. However, without the presence of cytosolic components, isolated cell nuclei displayed unimpeded DNA replication even in the presence of BTC.
6. Together with the continuous accelerated citrate export from the hepatoma’s cholesterol-enriched mitochondria, the loss of regulatory control at the HMGR locus of the cholesterogenesis pathway forecasts a potential accumulation of cholesterol, with consequential cholesterol enrichment of plasma membrane lipid rafts. Our exploratory studies with murine ascites hepatomas, *in vivo* and *in vitro*, revealed the rapid and continuous exfoliation of microvesicles, heavily enriched with cholesterol, from the tumor’s plasma membranes into the cell-free ascites fluid. Addition of mevalonate to the ascites culture medium dramatically increased the cholesterol content of the already-cholesterol-enriched exfoliated microvesicles. It is clearly recognized that microvesicle shedding from tumor plasma membranes commensurate with deregulated cholesterogenesis has provocative implications regarding the role of these processes in cancer immunosurveillance.
7. Finally, speculations are raised about potential regulated coordination between the CTP and PMCT in tumors.

## Author Contributions

All original research documented in this review was performed in the laboratory of PSC by research associates accredited in the references herein, in the Laboratory of Biochemistry, Department of Biology, New York University. RAP and many others performed the research. PSC and RAP contributed equally to the writing of this review, and both approved the submitted version.

## Conflict of Interest

The authors declare that the research was conducted in the absence of any commercial or financial relationships that could be construed as a potential conflict of interest.

## References

[B1] Abdel-Haleem A. M. Lewis N. E. Jamshidi N. Mineta K. Gao X. Gojobori T. (2017). The emerging facets of non-cancerous warburg effect. *Front. Endocrinol.* 8:279. 10.3389/fendo.2017.00279 29109698 PMC5660072

[B2] Aisenberg A. C. (1961). Studies on normal and neoplastic mitochondria. *I. Respirat. Cancer Res.* 21 295–303. 13681828

[B3] Aluvila S. Kotaria R. Sun J. Mayor J. A. Walters D. E. Harrison D. H. (2010). The yeast mitochondrial citrate transport protein: molecular determinants of its substrate specificity. *JBC* 285 27314–27326 10.1074/jbc.M110.137364 20551333 PMC2930730

[B4] Azrolan N. I. Coleman P. S. (1989). A discoordinate increase in the cellular amount of 3-hydroxy-3-methylglutaryl-CoA reductase results in the loss of rate-limiting control over cholesterogenesis in a tumour cell- free system. *Biochem. J.* 258 421–425. 10.1042/bj2580421 2705993 PMC1138379

[B5] Bao J. Zhu L. Zhu Q. Su J. Liu M. Huang W. (2016). SREBP-1 is an independent prognostic marker and promotes invasion and migration in breast cancer. *Oncol. Lett.* 12 2409–2416. 10.3892/ol.2016.4988 27703522 PMC5038874

[B6] Baulies A. Montero J. Matías N. Insausti N. Terrones O. Basañez G. (2018). The 2-oxoglutarate carrier promotes liver cancer by sustaining mitochondrial GSH despite cholesterol loading. *Redox Biol.* 14 164–177. 10.1016/j.redox.2017.08.022 28942194 PMC5609874

[B7] Bender T. Martinou J. C. (2016). The mitochondrial pyruvate carrier in health and disease: to carry or not to carry? *Biochimica et Biophysica Acta* 1863 2436–2442. 10.1016/j.bbamcr.2016.01.017 26826034

[B8] Bloch K. (1965). The biological synthesis of cholesterol. *Science* 150(3692), 19–28. 10.1126/science.150.3692.19 5319508

[B9] Brown M. S. Goldstein J. L. (1980). Multivalent feedback regulation of HMG CoA reductase, a control mechanism coordinating isoprenoid synthesis and cell growth. *J. Lipid Res.* 21 505–517. 6995544

[B10] Cairns R. A. Harris I. S. Mak T. W. (2011). Regulation of cancer cell metabolism. *Nat. Rev. Cancer* 11 85–95. 10.1038/nrc2981 21258394

[B11] Camara A. Zhou Y. Wen P. C. Tajkhorshid E. Kwok W. M. (2017). Mitochondrial VDAC1: a key gatekeeper as potential therapeutic target. *Front. Physiol.* 8:460. 10.3389/fphys.2017.00460 28713289 PMC5491678

[B12] Campbell A. M. Chan S. H. (2008). Mitochondrial membrane cholesterol, the voltage dependent anion channel (VDAC), and the warburg effect. *J. Bioenerget. Biomembranes* 40 193–197. 10.1007/s10863-008-9138-x 18677555

[B13] Carter H. Chen S. Isik L. Tyekucheva S. Velculescu V. E. Kinzler K. W. (2009). Cancer-specific high- throughput annotation of somatic mutations: computational prediction of driver missense mutations. *Cancer Res.* 69 6660–6667. 10.1158/0008-5472.CAN-09-1133 19654296 PMC2763410

[B14] Casey P. J. (1992). Biochemistry of protein prenylation. *J. Lipid. Res.* 33 1731–1740. 1479283

[B15] Casey P. J. (1995). Protein lipidation in cell signaling. *Science* 268 221–225. 10.1126/science.7716512 7716512

[B16] Cassim S. Vučetić M. Ždralević M. Pouyssegur J. (2020). Warburg and beyond: the power of mitochondrial metabolism to collaborate or replace fermentative glycolysis in cancer. *Cancers* 12 1119–1140. 10.3390/cancers12051119 32365833 PMC7281550

[B17] Catalina-Rodriguez O. Kolukula V. K. Tomita Y. Preet A. Palmieri F. Wellstein A. (2012). The mitochondrial citrate transporter, CIC, is essential for mitochondrial homeostasis. *Oncotarget* 3 1220–1235. 10.18632/oncotarget.714 23100451 PMC3717962

[B18] Chan S. H. Barbour R. L. (1983). Adenine nucleotide transport in hepatoma mitochondria. characterization of factors influencing the kinetics of ADP and ATP uptake. *Biochim. et Biophys. Acta* 723 104–113 10.1016/0005-2728(83)90014-2 6830767

[B19] Chaouch A. Porcelli V. Cox D. Edvardson S. Scarcia P. De Grassi A. (2014). Mutations in the mitochondrial citrate carrier SLC25A1 are associated with impaired neuromuscular transmission. *J. Neuromuscular Dis.* 1 75–90. 10.3233/JND-140021 26870663 PMC4746751

[B20] Chen H. W. Kandutsch A. A. Heiniger H. J. (1978). “The role of cholesterol in malignancy,” in *Membrane Anomalies of Tumor Cells. Prog Tumor Res.* ed. Wallach D. F. (Boston, MA: Basel, Karger Press), 22 275–316. 10.1159/000401203 209495

[B21] Chen H. W. Heiniger H. J. Kandutsch A. A. (1975). Relationship between sterol synthesis and DNA synthesis in phytohemagglutinin- stimulated mouse lymphocytes. *PNAS (USA)* 72 1950–1954. 10.1073/pnas.72.5.1950 1057774 PMC432666

[B22] Cluntun A. A. Lukey M. J. Cerione R. A. Locasale J. W. (2017). Glutamine metabolism in cancer: understanding the heterogeneity. *Trends Cancer* 3 169–180. 10.1016/j.trecan.2017.01.005 28393116 PMC5383348

[B23] Colell A. García-Ruiz C. Lluis J. M. Coll O. Mari M. Fernández- Checa J. C. (2003). Cholesterol impairs the adenine nucleotide translocator-mediated mitochondrial permeability transition through altered membrane fluidity. *JBC* 2278 33928–33935. 10.1016/j.redox.2019.101214 12821666

[B24] Coleman P. S. Chen L. C. Sepp-Lorenzino L. (1997). Cholesterol metabolism and tumor cell proliferation. *Subcell. Biochem.* 28 363–435. 10.1007/978-1-4615-5901-6_13 9090301

[B25] Coleman P. S. Ewell A. J. Good R. A. (1978a). Retention of susceptibility to mitogens after direct dansylation of viable human lymphocytes. *PNAS (USA)* 75 3766–3770. 10.1073/pnas.75.8.3766 278987 PMC392867

[B26] Coleman P. S. Lavietes B. Born R. Weg A. (1978b). Cholesterol enrichment of normal mitochondria *in vitro*: – A model system with properties of hepatoma mitochondria. *BBRC* 84 202–207. 10.1016/0006-291X90282-6 153138

[B27] Coleman P. S. Lavietes B. B. (1981). Membrane cholesterol, tumorigenesis, and the biochemical phenotype of neoplasia. *CRC Crit. Rev. Biochem.* 11 341–393. 10.1080/10409238109104421 6118236

[B28] Coleman P. S. Sepp-Lorenzino L. (1990). “The role of the cholesterol synthesis pathway during tumor cell proliferation,” in *Adv. Cholesterol Res.* eds Esfahani M. Swaney J. (New Jersey, NJ: Telford Press), 201–270.

[B29] Colombini M. (2004). VDAC: the channel at the interface between mitochondria and the cytosol. *Mol. Cell Biochem.* 256-257 107–115. 10.1023/b:mcbi.0000009862.17396.8d 14977174

[B30] Cox D. L. Nelson M. M. (2005). *Lehninger Principles of Biochemistry.* New York, NY: W.H. Freeman

[B31] Crabtree H. G. (1929). Observations on the carbohydrate metabolism of tumors. *Biochem. J.* 23 536–545. 10.1042/bj0230536 16744238 PMC1254097

[B32] Crain R. C. Clark R. W. Harvey B. E. (1983). Role of lipid transfer proteins in the abnormal lipid content of Morris hepatoma mitochondria and microsomes. *Cancer Res.* 43 3197–3202. 6850630

[B33] Cui J. Shi M. Xie D. Wei D. Jia Z. Zheng S. (2014). FOXM1 promotes the warburg effect and pancreatic cancer progression via transactivation of LDHA expression. *Clin. Cancer Res.* 20 2595–2606. 10.1158/1078-0432.CCR-13-2407 24634381 PMC4024335

[B34] DeBerardinis R. J. Chandel N. S. (2016). Fundamentals of cancer metabolism. *Sci. Adv.* 2 1–18. 10.1126/sciadv.1600200 27386546 PMC4928883

[B35] DeBerardinis R. J. Mancuso A. Daikhin E. Nissim I. Yudkoff M. Wehrli S. (2007). Beyond aerobic glycolysis: transformed cells can engage in glutamine metabolism that exceeds the requirement for protein and nucleotide synthesis. *PNAS (USA)* 104 19345–19350. 10.1073/pnas.0709747104 18032601 PMC2148292

[B36] Demel R. A. De Kruyff B. (1976). The function of sterols in membranes. *Biochim. Biophys. Acta* 457 109–132. 10.1016/0304-415790008-3 184844

[B37] Dietzen D. J. Davis E. J. (1993). Oxidation of pyruvate, malate, citrate, and cytosolic reducing equivalents by AS-30D hepatoma mitochondria. *Arch. Biochem. Biophys.* 305 91–102. 10.1006/abbi.1993.1397 8342959

[B38] Dietzen D. J. Davis E. J. (1994). Excess membrane cholesterol is not responsible for metabolic and bioenergetic changes in AS-30D hepatoma mitochondria. *Arch. Biochem. Biophys.* 309 341–347. 10.1006/abbi.1994.1122 8135546

[B39] Doyle J. W. Kandutsch A. A. (1988). Requirement for mevalonate in cycling cells: quantitative and temporal aspects. *J. Cell. Physiol.* 137 133–140. 10.1002/jcp.1041370116 3170653

[B40] Elstrom R. L. Bauer D. E. Buzzai M. Karnauskas R. Harris M. H. Plas D. R. (2004). Akt stimulates aerobic glycolysis in cancer cells. *Cancer Res.* 64 3892–3899. 10.1158/0008-5472.CAN-03-2904 15172999

[B41] Elustondo P. Martin L. A. Kaarten B. (2017). Mitochondrial cholesterol import. *Biochim. Biophys. Acta. Mol. Cell Biol. Lipids* 1862 90–101 10.1016/j.bbalip.2016.08.012 27565112

[B42] Epand R. M. (2006). Cholesterol and the interaction of proteins with membrane domains. *Prog. Lipid Res.* 45 279–294. 10.1016/j.plipres.2006.02.001 16574236

[B43] Erickson J. W. Cerione R. A. (2010). Glutaminase: a hot spot for regulation of cancer cell metabolism? *Oncotarget* 1 734–740. 10.18632/oncotarget.208 21234284 PMC3018840

[B44] Erickson S. K. Cooper A. D. Barnard G. F. Havel C. M. Watson J. A. Feingold K. R. Moser M. H. F. (1988). Regulation of cholesterol metabolism in a slow-growing hepatoma *in vivo*. *Biochim. et Biophys. Acta.* 960 131–138. 10.1016/0005-276090058-6 2835108

[B45] Fairbanks K. P. Witte L. D. Goodman D. S. (1984). Relationship between mevalonate and mitogenesis in human fibroblasts stimulated with platelet-derived growth factor. *JBC* 259 1546–1551. 6693424

[B46] Faubert B. Solmonson A. DeBerardinis R. J. (2020). Metabolic reprogramming and cancer progression. *Science* 368 1–22. 10.1126/science.aaw5473 32273439 PMC7227780

[B47] Feo F. Canuto R. A. Garcea R. Gabriel L. (1975). Effect of cholesterol content on some physical and functional properties of mitochondria isolated from adult rat liver, fetal liver, cholesterol- enriched liver and hepatomas AH-130, 3924A and 5123. *Biochimica et Biophysica Acta* 413 116–134. 10.1016/0005-273690063-2 172147

[B48] Flis V. V. Daum G. (2013). Lipid transport between the endoplasmic reticulum and mitochondria. *Cold Spring Harb. Perspect. Biol.* 5:a013235. 10.1101/cshperspect.a013235 23732475 PMC3660828

[B49] Fonsato V. Collino F. Herrera M. B. Cavallari C. Deregibus M. C. Cisterna B. (2012). Human liver stem cell-derived microvesicles inhibit hepatoma growth in SCID mice by delivering antitumor microRNAs. *Stem Cells* 30 1985–1998. 10.1002/stem.1161 22736596 PMC3468738

[B50] Frigerio F. Casimir M. Carobbio S. Maechler P. (2008). Tissue specificity of mitochondrial glutamate pathways and the control of metabolic homeostasis. *Biochim. Biophys. Acta* 1777 965–972. 10.1016/j.bbabio.2008.04.031 18486589

[B51] Fruman D. A. Rommel C. (2014). PI3K and cancer: lessons, challenges and opportunities. *Nat. Rev. Drug Discov.* 13 140–156. 10.1038/nrd4204 24481312 PMC3994981

[B52] Gnoni G. V. Priore P. Geelen M. J. Siculella L. (2009). The mitochondrial citrate carrier: metabolic role and regulation of its activity and expression. *IUBMB Life* 61 987–994. 10.1002/iub.249 19787704

[B53] Goldstein J. L. Brown M. S. (1990). Regulation of the mevalonate pathway. *Nature* 343 425–430. 10.1038/343425a0 1967820

[B54] Goldstein J. L. DeBose-Boyd R. A. Brown M. S. (2006). Protein sensors for membrane sterols. *Cell* 124 35–46. 10.1016/j.cell.2005.12.022 16413480

[B55] Grohsman J. Nowotny A. (1972). The immune recognition of TA3 tumors, its facilitation by endotoxin, and abrogation by ascites fluid. *J. Immunol.* 109 1090–1095. 4562425

[B56] Hanahan D. Weinberg R. A. (2011). Hallmarks of cancer: the next generation. *Cell* 144 646–674. 10.1016/j.cell.2011.02.013 21376230

[B57] Hanse E. A. Ruan C. Kachman M. Wang D. Lowman X. H. Kelekar A. (2017). Cytosolic malate dehydrogenase activity helps support glycolysis in actively proliferating cells and cancer. *Oncogene* 36 3915–3924. 10.1038/onc.2017.36 28263970 PMC5501748

[B58] Heiniger H. J. (1981). Cholesterol and its biosynthesis in normal and malignant. *Cancer Res.* 41(9 Pt 2), 3792–3794. 6973393

[B59] Hensley C. T. Wasti A. T. DeBerardinis R. J. (2013). Glutamine and cancer: cell biology, physiology, and clinical opportunities. The Journal of Clinical Investigation 123 3678–3684. 23999442 10.1172/JCI69600 PMC3754270

[B60] Hiller S. Abramson J. Mannella C. Wagner G. Zeth K. (2010). The 3D structures of VDAC represent a native conformation. *Trends Biochem. Sci.* 35 514–521. 10.1016/j.tibs.2010.03.005 20708406 PMC2933295

[B61] Holleran A. L. Fiskum G. Kelleher J. K. (1997). Quantitative analysis of acetoacetate metabolism in AS-30D hepatoma cells with 13C and 14C isotopic techniques. *Am. J. Physiol.* 272(6 Pt 1), E945–E951. 10.1152/ajpendo.1997.272.6.E945 9227436

[B62] Horvath S. E. Daum G. (2013). Lipids of mitochondria. *Prog. Lipid Res.* 52 590–614. 10.1016/j.plipres.2013.07.002 24007978

[B63] Hu F. Shi L. Min W. (2019). Biological imaging of chemical bonds by stimulated raman scattering microscopy. *Nat. Methods* 16 830–842. 10.1038/s41592-019-0538-0 31471618

[B64] Huard K. Brown J. Jones J. C. Cabral S. Futatsugi K. Gorgoglione M. (2015). Discovery and characterization of novel inhibitors of the sodium- coupled citrate transporter (NaCT or SLC13A5). *Sci. Rep.* 5:17391. 10.1038/srep17391 26620127 PMC4664966

[B65] Hulce J. J. Cognetta A. B. Niphakis M. J. Tully S. E. Cravatt B. F. (2013). Proteome-wide mapping of cholesterol-interacting proteins in mammalian cells. *Nat. Methods* 10 259–264. 10.1038/nmeth.2368 23396283 PMC3601559

[B66] Iacobazzi V. Infantino V. (2014). Citrate–new functions for an old metabolite. *Biol. Chem.* 395 387–399. 10.1515/hsz-2013-0271 24445237

[B67] Icard P. Poulain L. Lincet H. (2012). Understanding the central role of citrate in the metabolism of cancer cells. *Biochimica et Biophysica Acta* 1825 111–116. 10.1016/j.bbcan.2011.10.007 22101401

[B68] Jiang L. Boufersaoui A. Yang C. Ko B. Rakheja D. Guevara G. (2017). Quantitative metabolic flux analysis reveals an unconventional pathway of fatty acid synthesis in cancer cells deficient for the mitochondrial citrate transport protein. *Metab. Eng.* 43(Pt B), 198–207. 10.1016/j.ymben.2016.11.004 27856334 PMC5429990

[B69] Johnson B. M. DeBose-Boyd R. A. (2018). Underlying mechanisms for sterol-induced ubiquitination and ER-associated degradation of HMG CoA reductase. *Sem. Cell Dev. Biol.* 81 121–128. 10.1016/j.semcdb.2017.10.019 29107682 PMC6341991

[B70] Kaplan R. S. Morris H. P. Coleman P. S. (1982). Kinetic characteristics of citrate influx and efflux with mitochondria from morris hepatomas 3924A and 16. *Cancer Res.* 42 4399–4407. 7127281

[B71] Kaplan R. S. Parlo R. A. Coleman P. S. (1986). Measurement of citrate transport in tumor mitochondria. *Methods Enzymol.* 125 671–691. 10.1016/s0076-687925055-7 3520234

[B72] Kaplan R. S. (2001). Structure and function of mitochondrial anion transport proteins. *J. Membr. Biol.* 179 165–183. 10.1007/s002320010046 11246418

[B73] Kato T. Fahrmann J. F. Hanash S. M. Vykoukal J. (2020). Extracellular vesicles mediate B cell immune response and are a potential target for cancer therapy. *Cells* 9:1518. 10.3390/cells9061518 32580358 PMC7349483

[B74] Kennedy B. E. Madreiter C. T. Vishnu N. Malli R. Graier W. F. Karten B. (2014). Adaptations of energy metabolism associated with increased levels of mitochondrial cholesterol in Niemann-Pick type C1- deficient cells. *J. Biol. Chem.* 289 16278–16289. 10.1074/jbc.M114.559914 24790103 PMC4047397

[B75] Khatibzadeh N. Spector A. A. Brownell W. E. Anvari B. (2013) Effects of plasma membrane cholesterol level and cytoskeleton F-actin on cell protrusion mechanics. *PLoS ONE* 8:e57147. 10.1371/journal.pone.0057147 23451167 PMC3579816

[B76] Kolukula V. K. Sahu G. Wellstein A. Rodriguez O. C. Preet A. Iacobazzi V. (2014). SLC25A1, or CIC, is a novel transcriptional target of mutant p53 and a negative tumor prognostic marker. *Oncotarget* 5 1212–1225. 10.18632/oncotarget.1831 24681808 PMC4012738

[B77] Koppenol W. H. Bounds P. L. Dang C. V. (2011). Otto Warburg’s contributions to current concepts of cancer metabolism. *Nat. Rev. Cancer* 11 325–337. 10.1038/nrc3038 21508971

[B78] Langan T. J. Volpe J. J. (1987). Cell cycle-specific requirement for mevalonate, but not for cholesterol, for DNA synthesis in glial primary cultures. *J. Neurochem.* 49 513–521. 10.1111/j.1471-4159.1987.tb02894.x 3648095

[B79] LaNoue K. F. Schoolwerth A. C. (1979). Metabolite transport in mitochondria. *Annu. Rev. Biochem.* 48 871–922. 10.1146/annurev.bi.48.070179.004255 38739

[B80] Litvinov D. Y. Savushkin E. V. Dergunov A. D. (2018). Intracellular and plasma membrane events in cholesterol transport and homeostasis. *J. Lipids* 2018:3965054. 10.1155/2018/3965054 30174957 PMC6106919

[B81] Liu J. Rone M. B. Papadopoulos V. (2006). Protein-protein interactions mediate mitochondrial cholesterol transport and steroid biosynthesis. *JBC* 281 38879–38893. 10.1074/jbc.M608820200 17050526

[B82] Liu S. L. Sheng R. Jung J. H. Wang L. Stec E. O’Connor M. J. (2017). Orthogonal lipid sensors identify transbilayer asymmetry of plasma membrane cholesterol. *Nat. Chem. Biol.* 13 268–274. 10.1038/nchembio.2268 28024150 PMC5912897

[B83] Lu J. Tan M. Cai Q. (2015). The Warburg effect in tumor progression: mitochondrial oxidative metabolism as an anti-metastasis mechanism. *Cancer Lett.* 356(2 Pt A), 156–164. 10.1016/j.canlet.2014.04.001 24732809 PMC4195816

[B84] Luke J. C. Kaplan J. I. (1979). Theoretical shapes of bilipid vesicles under conditions of increasing membrane area. *Biophys. J.* 25 107–111. 10.1016/s0006-349585280-7 262380 PMC1328450

[B85] Maltese W. A. Sheridan K. M. (1985). Differentiation of neuroblastoma cells induced by an inhibitor of mevalonate synthesis: relation of neurite outgrowth and acetylcholinesterase activity to changes in cell proliferation and blocked isoprenoid synthesis. *J. Cell. Physiol.* 125 540–558. 10.1002/jcp.1041250326 3851809

[B86] Manna P. R. Ahmed A. U. Vartak D. Molehin D. Pruitt K. (2019). Overexpression of the steroidogenic acute regulatory protein in breast cancer: regulation by histone deacetylase inhibition. *Biochem. Biophys. Res. Commun.* 509 476–482. 10.1016/j.bbrc.2018.12.145 30595381 PMC6608577

[B87] Marquardt D. Kučerka N. Wassall S. R. Harroun T. A. Katsaras J. (2016). Cholesterol’s location in lipid bilayers. *Chem. Phys. Lipids* 199 17–25. 10.1016/j.chemphyslip.2016.04.001 27056099

[B88] Martin L. A. Kennedy B. E. Karten B. (2016). Mitochondrial cholesterol: mechanisms of import and effects on mitochondrial function. *J. Bioenerget. Biomembranes* 48 137–151. 10.1007/s10863-014-9592-6 25425472

[B89] Matés J. M. Campos-Sandoval J. A. Santos-Jiménez J. L. Márquez J. (2019). Dysregulation of glutaminase and glutamine synthetase in cancer. *Cancer Lett.* 467 29–39. 10.1016/j.canlet.2019.09.011 31574293

[B90] Mishra D. Banerjee D. (2019). Lactate dehydrogenases as metabolic links between tumor and stroma in the tumor microenvironment. *Cancers* 11:750. 10.3390/cancers11060750 31146503 PMC6627402

[B91] Mollinedo F. Fern M. Hornillos V. Delgado J. Amat-Guerri F. Acu A. U. (2011). Involvement of lipid rafts in the localization and dysfunction effect of the antitumor ether phospholipid edelfosine in mitochondria. *Cell Death Dis.* 2 1–9. 10.1038/cddis.2011.41 21593790 PMC3122120

[B92] Mondal M. Mesmin B. Mukherjee S. Maxfield F. R. (2009). Sterols are mainly in the cytoplasmic leaflet of the plasma membrane and the endocytic recycling compartment in CHO cells. *Mol. Biol. Cell* 20 581–588. 10.1091/mbc.e08-07-0785 19019985 PMC2626560

[B93] Mountford C. E. Wright L. C. Holmes K. T. Mackinnon W. B. Gregory P. Fox R. M. (1984). High-resolution proton nuclear magnetic resonance analysis of metastatic cancer cells. *Science* 226 1415–1418. 10.1126/science.6505699 6505699

[B94] Mullen A. R. Hu Z. Shi X. Jiang L. Boroughs L. K. Kovacs Z. (2014). Oxidation of alpha-ketoglutarate is required for reductive carboxylation in cancer cells with mitochondrial defects. *Cell Rep.* 7 1679–1690. 10.1016/j.celrep.2014.04.037 24857658 PMC4057960

[B95] Murtola T. J. Syvälä H. Pennanen P. Bläuer M. Solakivi T. Ylikomi T. (2012). The importance of LDL and cholesterol metabolism for prostate epithelial cell growth. *PLoS ONE* 7:e39445. 10.1371/journal.pone.0039445 22761797 PMC3384647

[B96] Mycielska M. E. Mohr M. Schmidt K. Drexler K. Rümmele P. Haferkamp S. (2019). Potential use of gluconate in cancer therapy. *Front. Oncol.* 9:522. 10.3389/fonc.2019.00522 31275855 PMC6593216

[B97] Mycielska M. E. Patel A. Rizaner N. Mazurek M. P. Keun H. Patel A. (2009). Citrate transport and metabolism in mammalian cells: prostate epithelial cells and prostate cancer. *BioEssays* 31 10–20. 10.1002/bies.080137 19153992

[B98] Mycielska M. E. Dettmer K. Rümmele P. Schmidt K. Prehn C. Milenkovic V. M. (2018). Extracellular citrate affects critical elements of cancer cell metabolism and supports cancer development *in vivo*. *Cancer Res.* 78 2513–2523. 10.1158/0008-5472.CAN-17-2959 29510993

[B99] Nie R. Stark S. Symersky J. Kaplan R. S. Lu M. (2017). Structure and function of the divalent anion/Na^+^ symporter from *Vibrio cholerae* and a humanized variant. *Nat. Commun.* 8:15009. 10.1038/ncomms15009 28436435 PMC5413979

[B100] Otto A. M. (2016). Warburg effect(s)-A biographical sketch of Otto Warburg and his impacts on tumor metabolism. *Cancer Metab.* 4:5. 10.1186/s40170-016-0145-9 26962452 PMC4784299

[B101] Pajor A. M. de Oliveira C. A. Song K. Huard K. Shanmugasundaram V. Erion D. M. (2016). Molecular basis for inhibition of the Na+/Citrate transporter NaCT (SLC13A5) by Dicarboxylate inhibitors. *Mol. Pharmacol.* 90 755–765. 10.1124/mol.116.105049 27683012

[B102] Palmieiri F. Stipani I. Quagliariello E. Klingenberg M. (1972). Kinetic study of the tricarboxylate carrier in rat liver mitochondria. *Eur. J. Biochem.* 26 587–594. 10.1111/j.1432-1033.1972.tb01801.x 5025933

[B103] Palmieri F. Monné M. (2016). Discoveries, metabolic roles and diseases of mitochondrial carriers: a review. *Biochimica et Biophysica Acta* 1863 2362–2378. 10.1016/j.bbamcr.2016.03.007 26968366

[B104] Palmieri F. (2013). The mitochondrial transporter family SLC25: identification, properties and physiopathology. *Mol. Asp. Med.* 34 465–484. 10.1016/j.mam.2012.05.005 23266187

[B105] Parlo R. A. Coleman P. S. (1986). Continuous pyruvate carbon flux to newly synthesized cholesterol and the suppressed evolution of pyruvate- generated CO2 in tumors: further evidence for a persistent truncated Krebs cycle in hepatomas. *Biochim. et Biophys. Acta* 886 169–176. 10.1016/0167-488990134-5 3083871

[B106] Parlo R. A. Coleman PS. (1984). Enhanced rate of citrate export from cholesterol-rich hepatoma mitochondria. the truncated Krebs cycle and other metabolic ramifications of mitochondrial membrane cholesterol. *J. Biol. Chem.* 259 9997–10003. 6469976

[B107] Pascale R. M. Calvisi D. F. Simile M. M. Feo C. F. Feo F. (2020). The Warburg effect 97 years after its discovery. *Cancers* 12 2819–2851. 10.3390/cancers12102819 33008042 PMC7599761

[B108] Pfrieger F. W. Vitale N. (2018). Cholesterol and the journey of extracellular vesicles. *J. Lipid Res.* 59 2255–2261. 10.1194/jlr.R084210 29678958 PMC6277151

[B109] Pike L. J. (2003). Lipid rafts: bringing order to chaos. *J. Lipid Res.* 44 655–667. 10.1194/jlr.R200021-JLR200 12562849

[B110] Poolsri W. A. Phokrai P. Suwankulanan S. Phakdeeto N. Phunsomboon P. Pekthong D. (2018). Combination of mitochondrial and plasma membrane citrate transporter inhibitors inhibits *de novo* lipogenesis pathway and triggers apoptosis in hepatocellular carcinoma cells. *BioMed. Res. Int.* 2018:3683026. 10.1155/2018/3683026 29546056 PMC5818947

[B111] Porstmann T. Griffiths B. Chung Y. L. Delpuech O. Griffiths J. R. Downward J. (2005). PKB/Akt induces transcription of enzymes involved in cholesterol and fatty acid biosynthesis via activation of SREBP. *Oncogene* 24 6465–6481. 10.1038/sj.onc.1208802 16007182

[B112] Potter M. Newport E. Morten K. J. (2016). The Warburg effect: 80 years on. *Biochem. Soc. Trans.* 44 1499–1505. 10.1042/BST20160094 27911732 PMC5095922

[B113] Puma P. (1982). *Vesicle Exfoliation from the Plasma Membrane of an Ascites Hepatoma.* thesis, Ph.D. dissertation/doctoral. New York University: New York, NY.

[B114] Quesney-Huneeus V. Galick H. A. Siperstein M. D. Erickson S. K. Spencer T. A. Nelson J. A. (1983). The dual role of mevalonate in the cell cycle. *JBC* 258 378–385. 6848509

[B115] Rahman M. M. Kipreos E. T. (2010). The specific roles of mitotic cyclins revealed. *Cell Cycle* 9 22–23. 10.4161/cc.9.1.10577 20016257

[B116] Rao S. Coleman P. S. (1989). Control of DNA replication and cell growth by inhibiting the export of mitochondrially derived citrate. *Exp. Cell. Res.* 180 341–352. 10.1016/0014-482790062-1 2492469

[B117] Raposo G. Stoorvogel W. (2013). Extracellular vesicles: exosomes, microvesicles, and friends. *J. Cell Biol.* 200 373–383. 10.1083/jcb.201211138 23420871 PMC3575529

[B118] Rauckhorst A. J. Taylor E. B. (2016). Mitochondrial pyruvate carrier function and cancer metabolism. *Curr. Opin. Genet. Dev.* 38 102–109. 10.1016/j.gde.2016.05.003 27269731 PMC5017534

[B119] Raz A. Goldman R. Yuli I. Inbar M. (1978). Isolation of plasma membrane fragments and vesicles from ascites fluid of lymphoma- bearing mice and their possible role in the escape mechanism of tumors from host immune rejection. *Cancer Immunol. Immunother* 4 53–59. 10.1007/BF00205571

[B120] Record M. Carayon K. Poirot M. Silvente-Poirot S. (2014). Exosomes as new vesicular lipid transporters involved in cell-cell communication and various pathophysiologies. *Biochim. Biophys. Acta* 1841 108–120. 10.1016/j.bbalip.2013.10.004 24140720

[B121] Reitzer L. J. Wice B. M. Kennell D. (1979). Evidence that glutamine, not sugar, is the major energy source for cultured HeLa cells. *J. Biol. Chem.* 254 2669–2676. 429309

[B122] Remani S. Sun J. Kotaria R. Mayor J. A. Brownlee J. M. Harrison D. H. (2008). The yeast mitochondrial citrate transport protein: identification of the Lysine residues responsible for inhibition mediated by Pyridoxal 5’- phosphate. *J. Bioenerg. Biomembr.* 40 577–585. 10.1007/s10863-008-9187-1w 19002576 PMC2775541

[B123] Repko E. M. Maltese W. A. (1989). Post-translational isoprenylation of cellular proteins is altered in response to mevalonate availability. *JBC* 264 9945–9952. 2722887

[B124] Ribas V. García-Ruiz C. Fernández-Checa J. C. (2016). Mitochondria, cholesterol and cancer cell metabolism. *Clin. Transl. Med.* 5:22. 10.1186/s40169-016-0106-5 27455839 PMC4960093

[B125] Rostovtseva T. K. Bezrukov S. M. (2008). VDAC regulation: role of cytosolic proteins and mitochondrial lipids. *J. Bioenerget. Biomembr.* 40 163–170. 10.1007/s10863-008-9145-y 18654841 PMC2671000

[B126] Sánchez-Martín C. C. Dávalos A. Martín-Sánchez C. de la Peña G. Fernández-Hernando C. Lasunción M. A. (2007). Cholesterol starvation induces differentiation of human leukemia HL-60 cells. *Cancer Res.* 67 3379–3386. 10.1158/0008-5472.CAN-06-4093? 17409448

[B127] Schneider H. Höchli M. Hackenbrock C. R. (1982). Relationship between the density distribution of intramembrane particles and electron transfer in the mitochondrial inner membrane as revealed by cholesterol incorporation. *J. Cell Biol.* 94 387–393. 10.1083/jcb.94.2.387 7107704 PMC2112900

[B128] Schroeder F. Huang H. McIntosh A. L. Atshaves B. P. Martin G. G. Kier A. B. (2010). Caveolin, sterol carrier protein-2, membrane cholesterol-rich microdomains and intracellular cholesterol trafficking. *Sub-Cellular Biochem.* 51 279–318. 10.1007/978-90-481-8622-8_10 20213548

[B129] Sebti S. M. (2005). Protein farnesylation: implications for normal physiology, malignant transformation, and cancer therapy. *Cancer Cell* 7 297–300. 10.1016/j.ccr.2005.04.005 15837619

[B130] Senyilmaz D. Teleman A. A. (2015). Chicken or the egg: warburg effect and mitochondrial dysfunction. *F1000Prime Reports* 7:41. 10.12703/P7-41 26097714 PMC4447048

[B131] Sepp-Lorenzino L. Rao S. Coleman P. S. (1991). Cell-cycle dependent, differential prenylation of proteins. *Eur. J. Biochem.* 200 579–590. 10.1111/j.1432-1033.1991.tb16221.x 1889420

[B132] Sepp-Lorenzino L. Rosen N. (1998). A farnesyl-protein transferase inhibitor induces p21 expression and G1 block in p53 wild type tumor cells. *JBC* 273 20243–20251. 10.1074/jbc.273.32.20243 9685373

[B133] Seyfried T. N. (2015). Cancer as a mitochondrial metabolic disease. *Front. Cell Dev. Biol.* 3:43. 10.3389/fcell.2015.00043 26217661 PMC4493566

[B134] Shoshan-Barmatz V. Ben-Hail D. Admoni L. Krelin Y. Tripathi S. S. (2015). The mitochondrial voltage-dependent anion channel 1 in tumor cells. *Biochim. Biophys. Acta* 1848(10 Pt B), 2547–2575. 10.3389/fonc.2017.00060 25448878

[B135] Sinensky M. Logel J. (1985). Defective macromolecule biosynthesis and cellcycle progression in a mammalian cell starved for mevalonate. *PNAS (USA)* 82, 3257–3261. 10.1073/pnas.82.10.3257 2582409 PMC397754

[B136] Singh P. Saxena R. Srinivas G. Pande G. Chattopadhyay A. (2013). Cholesterol biosynthesis and homeostasis in regulation of the cell cycle. *PLoS One* 8:e58833. 10.1371/journal.pone.0058833 23554937 PMC3598952

[B137] Siperstein M. D. Fagan V. M. (1964). Deletion of the cholesterol- negative feedback system in liver tumors. *Cancer Res.* 24 1108–1115. 14209404

[B138] Siperstein M. D. (1984). Role of cholesterogenesis and isoprenoid synthesis in DNA replication and cell growth. *J. Lipid Res.* 25 1462–1468. 6397554

[B139] Sola M. M. Oliver F. J. Salto R. Gutiérrez M. Vargas A. (1994). Citrate inhibition of rat-kidney cortex phosphofructokinase. *Mol. Cell. Biochem.* 135 123–128. 10.1007/BF00926514 7838139

[B140] Solsona-Vilarrasa E. Fucho R. Torres S. Nuñez S. Nuño-Lámbarri N. Enrich C. (2019). Cholesterol enrichment in liver mitochondria impairs oxidative phosphorylation and disrupts the assembly of respiratory supercomplexes. *Redox Biol.* 24 1–13. 10.1016/j.redox.2019.101214 31108462 PMC6526464

[B141] Sun J. Aluvila S. Kotaria R. Mayor J. A. Walters D. E. Kaplan R. S. (2010). Mitochondrial and plasma membrane citrate transporters: discovery of selective inhibitors and application to structure/function analysis. *Mol. Cell. Pharmacol.* 2 101–110. 20686672 PMC2913483

[B142] Tait S. W. Green D. R. (2012). Mitochondria and cell signalling. *J. Cell Sci.* 125(Pt 4), 807–815. 10.1242/jcs.099234 22448037 PMC3311926

[B143] Tamanoi F. Kato-Stankiewicz J. Jiang C. Machado I. Thapar N. (2001). Farnesylated proteins and cell cycle progression. *J. Cell. Biochem. Suppl.* 37 64–70. 10.1002/jcb.10067 11842430

[B144] Tolkach Y. Kristiansen G. (2018). The heterogeneity of prostate cancer: a practical approach. *Pathobiology* 85 108–116. 10.1159/000477852 29393241

[B145] Trams E. G. Lauter C. J. Salem N. Jr. Heine U. (1981). Exfoliation of membrane ecto-enzymes in the form of micro-vesicles. *Biochim. Biophys. Acta* 645 63–70. 10.1016/0005-273690512-5 6266476

[B146] Usenik A. Legiša M. (2010). Evolution of allosteric citrate binding sites on 6-phosphofructo-1-kinase. *PLoS One* 5:e15447. 10.1371/journal.pone.0015447 21124851 PMC2990764

[B147] Vacanti N. M. Divakaruni A. S. Green C. R. Parker S. J. Henry R. R. Ciaraldi T. P. (2014). Regulation of substrate utilization by the mitochondrial pyruvate carrier. *Mol. Cell* 56 425–435. 25458843 10.1016/j.molcel.2014.09.024 PMC4267523

[B148] Vámosi G. Bodnár A. Vereb G. Szöllösi J. Damjanovich S. (2006). Role of lipid microdomains in the formation of supramolecular protein complexes and transmembrane signaling. *Lipid Rafts and Caveolae* (Fielding C. J. ed) Wiley-Vch Verlag GmbH & Co: Hoboken, NJ. 10.1002/3527608079.ch7

[B149] van Blitterswijk W. J. Emmelot P. Hilkmann H. A. Hilgers J. Feltkamp C. A. (1979). Rigid plasma-membrane-derived vesicles, enriched in tumour-associated surface antigens (MLr), occurring in the ascites fluid of a murine leukaemia (GRSL). *Int. J. Cancer* 23 62–70. 10.1002/ijc.2910230112 83306

[B150] van Hoeven R. P. Emmelot P. (1972). Studies on plasma membranes : XVIII. lipid class composition of plasma membranes isolated from rat and mouse liver and hepatomas. *J. Membr. Biol.* 9 105–126. 10.1007/BF01868047 24177643

[B151] Vander Heiden M. G. Cantley L. C. Thompson C. B. (2010). Understanding the Warburg effect: the metabolic requirements of cell proliferation. *Science* 324(5930), 1029–1033. 10.1126/science.1160809 19460998 PMC2849637

[B152] Voet D. Voet J. G. (2011). *Biochemistry* 4th ed. New York, NY: John Wiley and Sons.

[B153] Vogelstein B. Papadopoulos N. Velculescu V. E. Zhou S. Diaz L. A. Jr. Kinzler K. W. (2013). Cancer genome landscapes. *Science* 339 1546–1558. 10.1126/science.1235122 23539594 PMC3749880

[B154] Vu L. T. Peng B. Zhang D. X. Ma V. Mathey-Andrews C. A. Lam C. K. (2019). Tumor-secreted extracellular vesicles promote the activation of cancer-associated fibroblasts via the transfer of microRNA-125b. *J. Extracell. Vesicles* 8:1599680. 10.1080/20013078.2019.1599680 31044053 PMC6484490

[B155] Wang D. Yin L. Wei J. Yang Z. Jiang G. (2017). ATP citrate lyase is increased in human breast cancer, depletion of which promotes apoptosis. *Tumour Biol.* 39:1010428317698338. 10.1177/1010428317698338 28443474

[B156] Wang M. Casey P. J. (2016). Protein prenylation: unique fats make their mark on biology. *Nat. Rev. Mol. Cell Biol.* 17 110–122. 10.1038/nrm.2015.11 26790532

[B157] Warburg O. (1925). The metabolism of carcinoma cells. *J. Cancer Res.* 9 148–163. 10.1158/jcr.1925.148

[B158] Warburg O. (1956). On the origin of cancer cells. *Science* 123 309–314. 10.1126/science.123.3191.309 13298683

[B159] Weiser B. P. Salari R. Eckenhoff R. G. Brannigan G. (2014). Computational investigation of cholesterol binding sites on mitochondrial VDAC. *J. Phys. Chem. B* 118 9852–9860. 10.1021/jp504516a 25080204 PMC4141696

[B160] Wise D. R. Ward P. S. Shay J. E. Cross J. R. Gruber J. J. Sachdeva U. M. (2011). Hypoxia promotes isocitrate dehydrogenase-dependent carboxylation of α-ketoglutarate to citrate to support cell growth and viability. *PNAS (USA)* 108 19611–19616. 10.1073/pnas.1117773108 22106302 PMC3241793

[B161] Yang C. Ko B. Hensley C. T. Jiang L. Wasti A. T. Kim J. (2014). Glutamine oxidation maintains the TCA cycle and cell survival during impaired mitochondrial pyruvate transport. *Mol. Cell.* 56 414–424. 10.1016/j.molcel.2014.09.025 25458842 PMC4268166

[B162] Zaidi N. Swinnen J. V. Smans K. (2012). ATP-citrate lyase: a key player in cancer metabolism. *Cancer Res.* 72 3709–3714. 10.1158/0008-5472.CAN-11-4112 22787121

